# Ecological Changes Exacerbating the Spread of Invasive Ticks has Driven the Dispersal of Severe Fever with Thrombocytopenia Syndrome Virus Throughout Southeast Asia

**DOI:** 10.1093/molbev/msae173

**Published:** 2024-08-28

**Authors:** Lester J Pérez, Guy Baele, Samuel L Hong, Gavin A Cloherty, Michael G Berg

**Affiliations:** Infectious Disease Research, Abbott Diagnostics Division, Abbott Laboratories, Abbott Park, IL, USA; Abbott Pandemic Defense Coalition (APDC), Abbott Park, IL, USA; Department of Microbiology, Immunology and Transplantation, Laboratory of Clinical and Evolutionary Virology, Rega Institute, KU Leuven, Leuven, Belgium; Department of Microbiology, Immunology and Transplantation, Laboratory of Clinical and Evolutionary Virology, Rega Institute, KU Leuven, Leuven, Belgium; Infectious Disease Research, Abbott Diagnostics Division, Abbott Laboratories, Abbott Park, IL, USA; Abbott Pandemic Defense Coalition (APDC), Abbott Park, IL, USA; Infectious Disease Research, Abbott Diagnostics Division, Abbott Laboratories, Abbott Park, IL, USA; Abbott Pandemic Defense Coalition (APDC), Abbott Park, IL, USA

**Keywords:** severe fever with thrombocytopenia syndrome virus, evolution, ecological, climatic factors, phylodynamic

## Abstract

Severe fever with thrombocytopenia syndrome virus (SFTSV) is a tick-borne virus recognized by the World Health Organization as an emerging infectious disease of growing concern. Utilizing phylodynamic and phylogeographic methods, we have reconstructed the origin and transmission patterns of SFTSV lineages and the roles demographic, ecological, and climatic factors have played in shaping its emergence and spread throughout Asia. Environmental changes and fluctuations in tick populations, exacerbated by the widespread use of pesticides, have contributed significantly to its geographic expansion. The increased adaptability of Lineage L2 strains to the *Haemaphysalis longicornis* vector has facilitated the dispersal of SFTSV through Southeast Asia. Increased surveillance and proactive measures are needed to prevent further spread to Australia, Indonesia, and North America.

## Introduction

Severe fever with thrombocytopenia syndrome virus (SFTSV) is a tick-borne *Phlebovirus* (Liu et al. 2014), first identified in China in 2009 (Yu et al. 2011), that has since rapidly spread to several neighboring East Asian countries (Takahashi et al. 2013; Kim et al. 2018; Tran et al. 2019; Win et al. 2020; Rattanakomol et al. 2022). Clinically, the virus induces an elevated and prolonged fever (22.5 ± 7.6 days) accompanied by thrombocytopenia (Luo et al. 2023), gastrointestinal distress (Akagi et al. 2020), and in severe cases, multiorgan failure (Li et al. 2018b). Biologically, as a bunyavirus, it is comprised of three genome segments, enabling it to reassort and rapidly acquire new genetic diversity. With a case fatality rate that varies from 16% (Li et al. 2018a) to 40% (Tian et al. 2020) and with confirmed cases of human-to-human transmission (Liu et al. 2012) through close contact or health workers via blood or bloody respiratory secretions (Jung et al. 2019), vomit (Ryu et al. 2018), and aerosols (Moon et al. 2019; Hu et al. 2022), SFTSV poses a significant public health threat. As such, it has garnered significant interest from clinical and epidemiological research communities (Kim and Park 2023).

Recent studies have provided invaluable insights into the phylogenetic relationships of SFTSV strains (Wang et al. 2023), sources of genetic diversity (Dai et al. 2022), and the dating of its emergence (Liu et al. 2016, 2021). Nevertheless, there are many open questions within this realm and still others that require revisiting due to methodological concerns. Specifically, prior attempts to determine the virus temporal origin have faced limitations, including the use of restricted datasets (Li et al. 2021) and the absence of hierarchical phylogenetic models, especially when concatenating viral segments with variable evolutionary rates (Liu et al. 2016). Similarly, the exclusive use of tanglegrams for pinpointing reassortant strains (in general; Li et al. 2021; Wang et al. 2023) has resulted in interpretations that could be potentially misleading (de Vienne 2019). Ecological factors that influence the transmission and spread of SFTSV also require additional investigation. While the tick species *Haemaphysalis longicornis* has been widely accepted as the principal vector for SFTSV (Casel et al. 2021), the role of other tick species, such as *Amblyomma testudinarium*, *Haemaphysalis flava*, and *Haemaphysalis hystricis* that have also been associated with SFTSV transmission (Sato et al. 2021), deserve further attention. The role of mammalian hosts in SFTSV ecology is also not fully understood. Cats have been identified as highly susceptible hosts (Park et al. 2019) with evidence for cat-to-cat nosocomial outbreaks in veterinary clinics and direct cat-to-human transmission of SFTSV (Yamanaka et al. 2020). Hedgehogs have also been suggested as intermediaries who serve to amplify SFTSV viral loads and facilitate transmission between ticks and humans (Zhao et al. 2022). Finally, there is a considerable gap in our understanding of the influence of human activities and environmental factors on the epidemiology of SFTSV. For instance, human interventions in agriculture and changes in land-use could alter tick habitats and consequently affect the spread of the virus. Similarly, climate variables like temperature and humidity could have a seasonal impact on tick activity, thereby influencing the rate of SFTSV transmission.

In this study, we employ a multidisciplinary approach that combines advanced viral phylodynamics, continuous phylogeography, and landscape phylogeographic methods to address many of these questions. This involves integrating genomic data with analytical and modeling methodologies to reveal the interactions between SFTSV and different climate variables and ecological factors. Through this framework, we intend to shed new light on the evolutionary history of SFTSV and provide an understanding of how natural and anthropogenic environmental changes may have influenced its transmission and geographical distribution. These insights should predict the likely trajectory of SFTSV spread, guide future research, and inform public health interventions.

## Results

### SFTSV Phylogenetic Structure and Mechanisms of Genetic Diversity

Prior to this study, Li et al. (2021) reported the presence of six genotypes for the M and L segments and five for the S segment. We analyzed all available sequences for the three segments of SFTSV (L *n* = 1,528, M *n* = 1,815, and S n = 2,009) by maximum likelihood (ML) reconstruction (supplementary fig. S1, Supplementary Material online). Examining the support for the internal nodes, only considering bootstrap values >70%, we determined that there are only three genetically distinct clades (Clades I to III). Pairwise sequence comparison (PASC) distributions further indicated that a genetic distance cutoff >2% would be needed for the establishment of genotypes within this viral species (supplementary fig. S1, Supplementary Material online). Fitting both parameters (bootstrap >70% and a genetic distance >2%) in cluster-picker analysis failed to identify monophyletic clusters across the phylogenies of all segments that would otherwise denote the presence of genotypes for SFTSV.

**Fig. 1.**
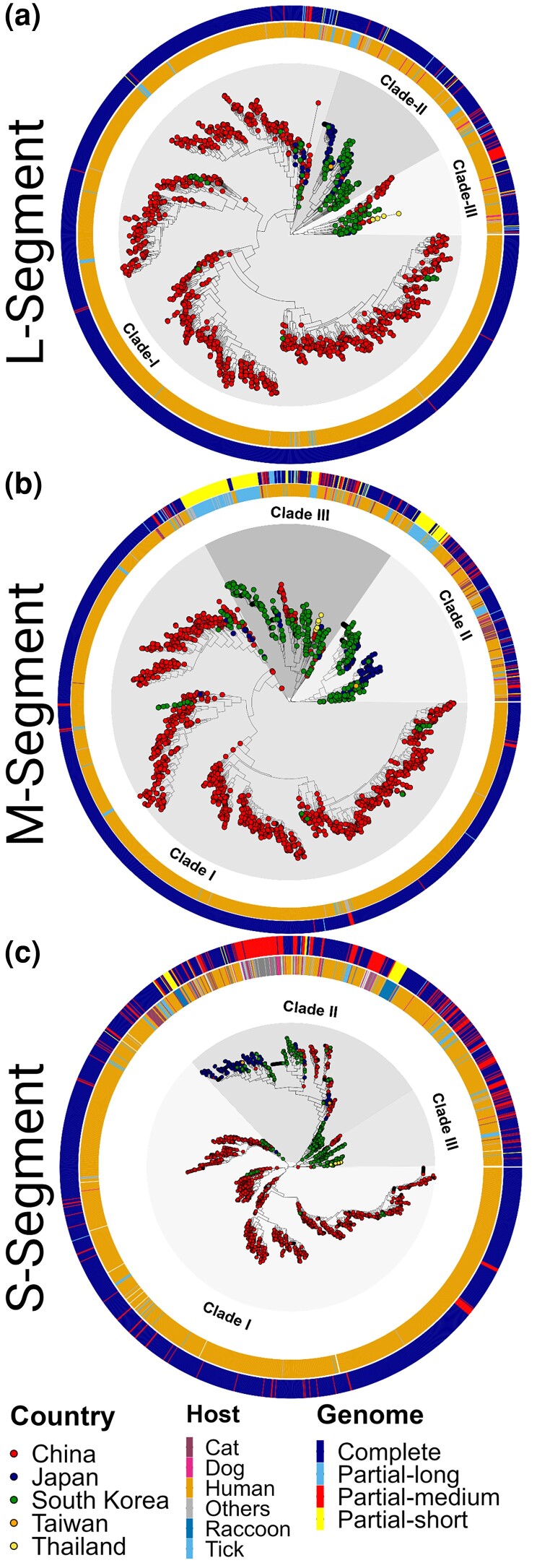
Phylogenetic analysis of SFTSV genomic segments. Maximum likelihood trees represent the phylogeny of all nonredundant SFTSV genomes in GenBank for segments a) L, b) M, and c) S. After the PASC and lineage demarcation (see supplementary fig. S1, Supplementary Material online), we identified three primary clades per segment. Location of the strain's isolation (country) is color coded at the tips, while the inner and outer circles indicate the isolation host and the influence of sequence length on phylogeny, respectively. To enhance the clarity of host range visualization in the figure, tick has been collectively labeled as “Tick.” The figure highlights host from which a higher frequency of sequences (*n* > 15) have been obtained, while less frequently sampled hosts are grouped under the label “Others.” For a comprehensive view of all hosts from which genomic data have been collected, please see supplementary fig. S2, Supplementary Material online. In all cases, the data were integrated using the *ggtreeExtra* R package.

**Fig. 2.**
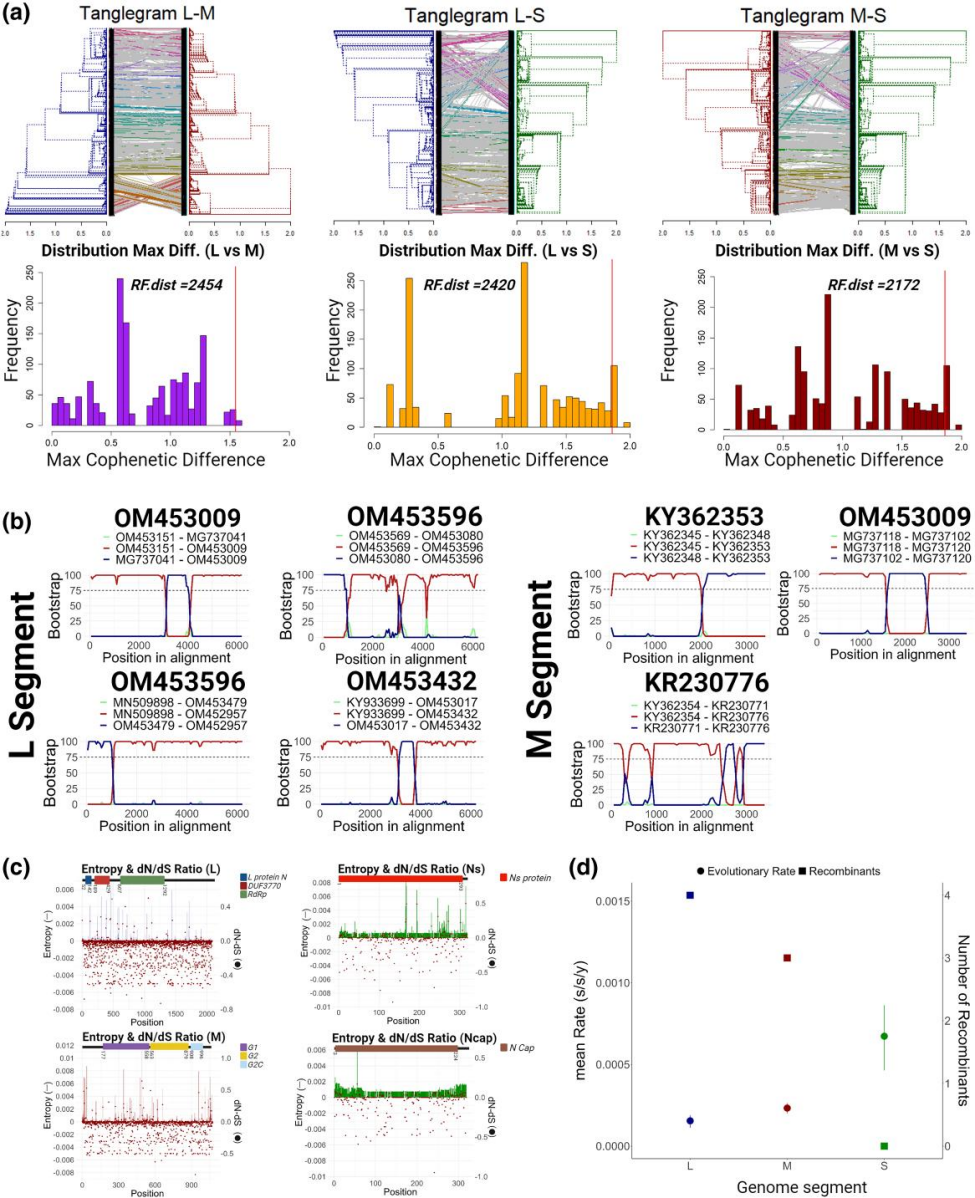
Mechanisms of SFTSV genetic diversity. a) Tanglegram comparison of maximum likelihood trees for genomic segments L vs. M (left), L vs. S (center), and M vs. S (right), highlighting topological incongruences (see supplementary fig. S3, Supplementary Material online for initial strain identification). Subsequent analysis using ultrametric trees and cophenetic distances computed by the *prophogorn* R package (PASC cutoff set at 2%, as detailed in supplementary fig. S1, Supplementary Material online) revealed no reassortant strains, though significant RF distances were noted. b) The detection of recombination breakpoints in L and M segments as determined by RDP5v5 and confirmed by the temporal evaluation of their parental strains (supplementary figs. S4 to S6, Supplementary Material online), visualized with Bootscan and presenting only those with clear signals and bootstrap values ≥75% (all recombinants found in the study are listed in supplementary fig. S4, Supplementary Material online and the temporal analysis of their parental strains are displayed in supplementary figs. S5 and S6, Supplementary Material online). c) Adaptive divergence assessed by nonsynonymous/synonymous rate differences (d*N*/d*S*) and amino acid entropy rates (Shannon entropy), mapped across three coding regions of annotated SFTSV proteins. Variability domains are indicated above the entropy bar graphs, with d*N*/d*S* ratios represented by dots. d) Comparative plot of the number of recombinant strains per segment against evolutionary rates estimated by BEASTv1.10.5, including the 95% HPD intervals.

We next explored the features driving the phylogenetic reconstruction of SFTSV by integrating the geographic location of sampling, host of isolation, and the sequence length analyzed with the phylogenies obtained (Fig. 1). Clustering of SFTSVs strains was clearly along geographical lines, with Clade I predominantly composed of sequences isolated from China (∼95%), and Clades II and III primarily composed of sequences from South Korea (∼60% and 56%, respectively). Clade II also contained sequences isolated from Japan (∼39%) and Clade III had sequences from China (∼32%) and Thailand (∼3%; Fig. 1). Despite the oversampling of sequences from humans, the host species trait was distributed across the tree and thus not a determinant of clade composition (Fig. 1). We observed, however, that a wide variety of hosts were concentrated in Clades II and III compared with Clade I. Similarly, the lack of individual clusters driven by sequence length indicated this factor apparently, does not impact the phylogenetic organization, suggesting the potential utility of partial sequences, particularly for laboratories with limited sequencing capacity (Fig. 1). These results suggest that either the evolution or the diversification of SFTSV is driven by its local geographical dispersion. We therefore examined the possible mechanisms of acquiring increased genetic diversity: reassortment (due to its segmented nature), homologous recombination, adaptive/selective divergence, and the evolutionary rate for each segment of the virus (Fig. 2). Previous studies have identified reassortants among different genotypes (Li et al. 2021; Dai et al. 2022; Wang et al. 2023); however, since our analysis did not sustain the presence of genetically distinct genotypes, we only considered reassortants as those strains whose sequences were located in different clades (I to III). An initial visualization using tanglegrams of the ML-reconstructed trees indeed highlighted several discrepancies between the segment topologies (Fig. 2a), suggesting reassortment could be a mechanism by which SFTSV diversifies (supplementary fig. S3, Supplementary Material online). However, considering that tanglegrams could mislead the link between the level of entanglement and the level of congruence between trees (de Vienne 2019), together with the fact that for two strains to exchange segments there is a need to coincide in time (Müller et al. 2020; Gong et al. 2021) we explored the validity of these reassortment events. Thus, we transformed the ML topologies into ultrametric trees to incorporate evolutionary time and then explored the distribution of the cophenetic distances between all the pair trees (Fig. 2a, bottom; supplementary fig. S1, Supplementary Material online). When applying the same PASC distribution cutoff value of 2% (Fig. 2a), this resulted in the lack of reassortment events. Our results suggest that previous identifications of SFTSV reassortants might have been overestimated due to methodological limitations. Nonetheless, we further examined if there were incongruences among the topologies by estimating the Robinson–Foulds (RF) distances. The elevated values obtained for the different pair of trees (Fig. 2) confirmed the presence of inconsistencies in the topological agreement among the segments, indicating we are either being too conservative (e.g. false negatives for reassortment) or other mechanisms may be involved.

A major source of topological disagreement among different viral segments is often homologous recombination. Thus, we reduced our dataset to include only complete-genome sequences for each segment and we identified 20 recombinants in Segment L, 8 in Segment M, and only 2 in Segment S (Fig. 2 and supplementary fig. S4, Supplementary Material online). For both L and M segments, different breakpoints in the genome were identified as hotspots for recombination (supplementary fig. S4, Supplementary Material online), whereas both recombinants for the S segment were identified in the same region upstream of the coding sequence for nucleocapsid (supplementary fig. S4, Supplementary Material online). Applying the same logic and stringency as for reassortment, for recombination to occur the two parental strains must have co-circulated during the same period and emerged from a common ancestor (supplementary figs. S4 and S5, Supplementary Material online). Consequently, of the 20 recombinants identified in the L segment, 4 were confirmed as true positives based on this temporal premise (Fig. 2 and supplementary figs. S4 and S5, Supplementary Material online). For the M segment, three recombinants were considered likely due to the co-temporal circulation of their parental strains (Fig. 2 and supplementary figs. S4 and S5, Supplementary Material online). However, both recombinants in the S segment were dismissed as false positives, since their parental strains neither shared a common ancestor nor co-circulated in the same epoch (supplementary fig. S6, Supplementary Material online). Thus, while recombination was observed with different frequencies for each segment, we hypothesized that the relative number and differences in breakpoint distributions were the consequence of additional evolutionary pressures on segments. By estimating the ratio of nonsynonymous to synonymous substitutions (d*N*/d*S*), we inferred the action (or lack) of selective pressure. The cumulative d*N*/d*S* ratios for the coding region of L, M, and S segments resulted in values of 0.0725831, 0.1567993, 0.2964018, and 0.2877241, respectively (S segment encodes for two proteins, Ns and nucleocapsid; Fig. 2c). This indicates the L segment is under a strong purifying selection, whereas M and S segments are likely subject to slightly more relaxed purifying selection or episodic positive selection. We further untangled the contribution of natural selection on a per codon basis using the method described by Nei and Gojobori (1986), wherein amino acid changes are denoted by the Shannon entropy (Fig. 2c).

We confirmed that most of the codon sites in the L segment are indeed under strong purifying selection; however, we also identified an N-terminal *DUF3770* domain of unknown function whose mutations were identified as possibly advantageous. Similarly, most mutations in the M segment also appear to be under negative selection, although amino acid replacements in the phlebovirus G1 and G2C domains also seem beneficial. We observed that the functional domains for RdRp (L segment) and phlebovirus G2 (M segment) are under strong purifying selection. In contrast, mutations in both translated products of the S segment are either neutral or positively selected, although there are several negatively selected mutations in the NS protein upstream of the region. We also estimated the evolutionary rates for each genomic segment, which yielded a mean value for the L segment of 1.5636 × 10^−4^ highest posterior density (HPD)95% (1.0202 × 10^−4^, 2.0529 × 10^−4^) substitution/site/year, for M segment of 3.5377 × 10^−4^ HPD95% (1.9989 × 10^−4^, 2.643 × 10^−4^), and for S segment 6.8095 × 10^−4^ HPD95% (4.6367 × 10^−4^, 8.6339 × 10^−4^). This demonstrates the S segment is evolving at least four times faster than the L segment and two times faster than the M segment and agrees with the natural selection pattern we identified. By plotting the evolutionary rates together with the number of recombinants detected, we can readily discern a pattern wherein higher evolutionary rates correspond to lower number of recombinants and vice versa (Fig. 2d). Together these results suggest that breakpoint distributions across the segments of SFTSV counteract the Error-Catastrophe phenomenon in the virus (Tejero et al. 2011; de Aguiar et al. 2015).

### Origin, Expansion, and Other Factors Associated with the Dispersal Trajectory of SFTSV

**Fig. 3.**
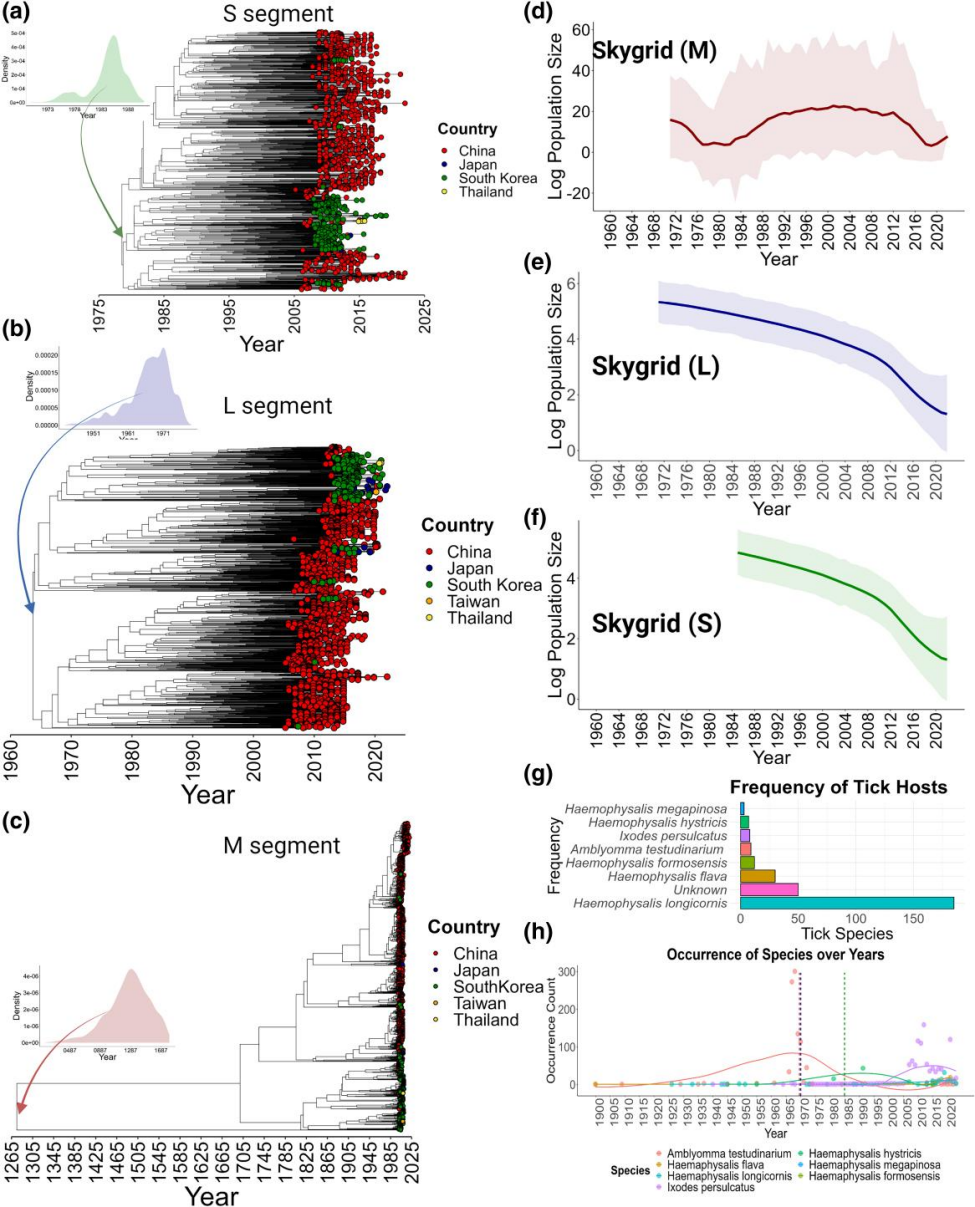
Tick–host dynamics and evolutionary demography of SFTSV genomic segments. a to c) Time-scaled MCC phylogenies for SFTSV genomic segments L, M, and S, with sampling countries indicated by the color of the tips. The 95% posterior density intervals for the MRCA of each segment are denoted: M-segment, L-segment, and S-segment. d to f) SkyGrid plots showing changes in the effective population size of SFTSV over time. g) Vector diversity analysis determined by the frequency of ticks identified in the genomic metadata (each tick species is denoted). h) The historical tick population dynamics from 1900, presented with occurrence data (dots) and trend lines from loess regression models. Ticks' occurrence, collection date and coordinates were obtained from the GBIF (see Materials and Methods). Vertical dotted lines indicate the inception of the demographic expansion for each segment (red for M, blue for L, and green for S) as estimated by Bayesian SkyGrid analysis.

Retrospective SFTSV+ samples that preceded the initial outbreak in China only date back to 2005 (Liu et al. 2012). To estimate the most-recent common ancestor (MRCA) for all three segments of SFTSV, we first evaluated the temporal signal in the genomic data in relation to the associated isolation dates. The refined sequence set with outliers removed exhibited coefficients of the root-to-tip regression of *R*^2^ = 0.8 for the L and M segment and *R*^2^ = 0.6 (supplementary fig. S7, Supplementary Material online) and robust evidence of a temporal signal, as confirmed by a Bayesian Evaluation of Temporal Signal (BETS) analysis (Duchene et al. 2020; see supplementary table S1, Supplementary Material online). An uncorrelated relaxed clock model with an underlying exponential distribution provided a superior fit to the data compared with a strict clock model (supplementary table S1, Supplementary Material online). Dating inferred from posterior densities and maximum clade credibility (MCC) trees revealed distinct emergence timelines for each SFTSV segment (Fig. 3a to c). The M segment emerged earliest in approximately the Year 1265 (HPD95% [726, 1766]; Fig. 3c), followed by the L segment around 1966 (HPD95% [1954, 1977]; Fig. 3b), and the S segment in 1985 (HPD95% [1975, 1989]; Fig. 3a). A similar sequential emergence of genomic segments has been observed in Oropouche (Gutierrez et al. 2020) and Bangui bunyaviruses (Orf et al. 2023), among others. Our results underscore the connection between the emergence of new species within the Bunyavirales order and genome reshuffling events occurring within their respective vectors.

To understand its evolutionary history and pinpoint when and how these events may have occurred for SFTSV, we analyzed genetic diversity patterns (Fig. 3d to f). Interestingly, while the tMRCA for the M segment dated back to 1265, the Bayesian SkyGrid reconstruction commenced only from 1970 (Fig. 3d), indicating a period of evolutionary stasis in its genetic diversity. A similar temporal starting point (1970) was observed for the L segment (Fig. 3e). This synchronized onset in the reconstructions of both segments suggests a potential shared evolutionary event or mechanism influencing these segments around the same period. The concurrent emergence or diversification of both M and L segments around 1970 could be indicative of a significant reassortment event that might have given rise to a lineage (or lineages) of SFTSV that possessed genomic segments better adapted to prevailing conditions or hosts. It is also possible that changes in one segment, such as the L segment, could have had downstream effects on the M segment or vice versa, signifying a functional interdependence wherein a change in one protein necessitates a complementary change in the other. The reconstruction for the S segment began later in 1985 (Fig. 3f), a delay in emergence which coincided in time with an uptick in the genetic diversity of the M segment (Fig. 3d and f). This suggests an event in which the M segment underwent diversification in response to, or in concert with, changes in the S segment, possibly due to a functional or evolutionary interplay between these two segments. The simultaneous evolutionary shifts in the M and S segments might have had implications for the virus' transmissibility, virulence, or host range, collectively enhancing the fitness of certain SFTSV lineages.

We next explored the ecological factors that may have impacted the genetic fitness of SFTSV and facilitated the genomic arrangement suggested by the SkyGrid reconstructions (Fig. 3d to f), turning first to the prevalence of tick vectors. *H. longicornis* is the most frequently reported source of virus isolation (Figs. 1 and 3g), aligning with the claim that this species is the main vector for SFTSV transmission (Dai et al. 2022), although there is potential for sampling bias. Using publicly available data for Asia, we performed a temporal dynamics analysis of tick populations in which SFTSV has been detected (Fig. 3h). The occurrence of *A. testudinarium* peaked at a maximum in 1970 (Fig. 3h). Comparing this to the SkyGrid reconstructions for M and L (Fig. 3d and e) segments suggests this tick species may have played a pivotal role at the ecological interface of the virus' first reassortment event. Steadily increased levels of *H. hystricis* between 1960 and 2010 (Fig. 3h) provide a timeframe during which these two tick species predominated. Notably, in 1985, both species exhibited equivalent levels of occurrence (Fig. 3h), aligning with the starting point of the S segment SkyGrid reconstruction. While perhaps a series of coincidences, the trends parallel the evolutionary milestones observed in the SFTSV segments and suggest *H. hystricis* may have influenced the genetic makeup of SFTSV strains that eventually went on to infect human hosts.

There are additional take-aways from the SkyGrid reconstructions. From 2012 to 2018, each segment displayed a decline in genetic diversity (Fig. 3d to f) reminiscent of a bottleneck effect. This trend may be attributed to the reduced occurrence of tick species, specifically *A. testudinarium* and *H. hystricis* (Fig. 3h), or alternatively, it could be linked to a potential host shift to *H. longicornis*. Here, we observe that the occurrence of *H. longicornis* began to rise around the Year 2000 and has increased up to the time of this study. In the last 4 years (2018 to 2022), there has been resurgence in genetic diversity, which could indicate adaptation to this new host, facilitating the wider dissemination of SFTSV across Asia. Thus, a consistent timeline has emerged that brings together the dating of genomic segments, population densities of known tick vectors, and trends in viral genetic diversity to explain the recency of human infections and the preponderance of SFTSV isolation from *H. longicornis*.

**Fig. 4.**
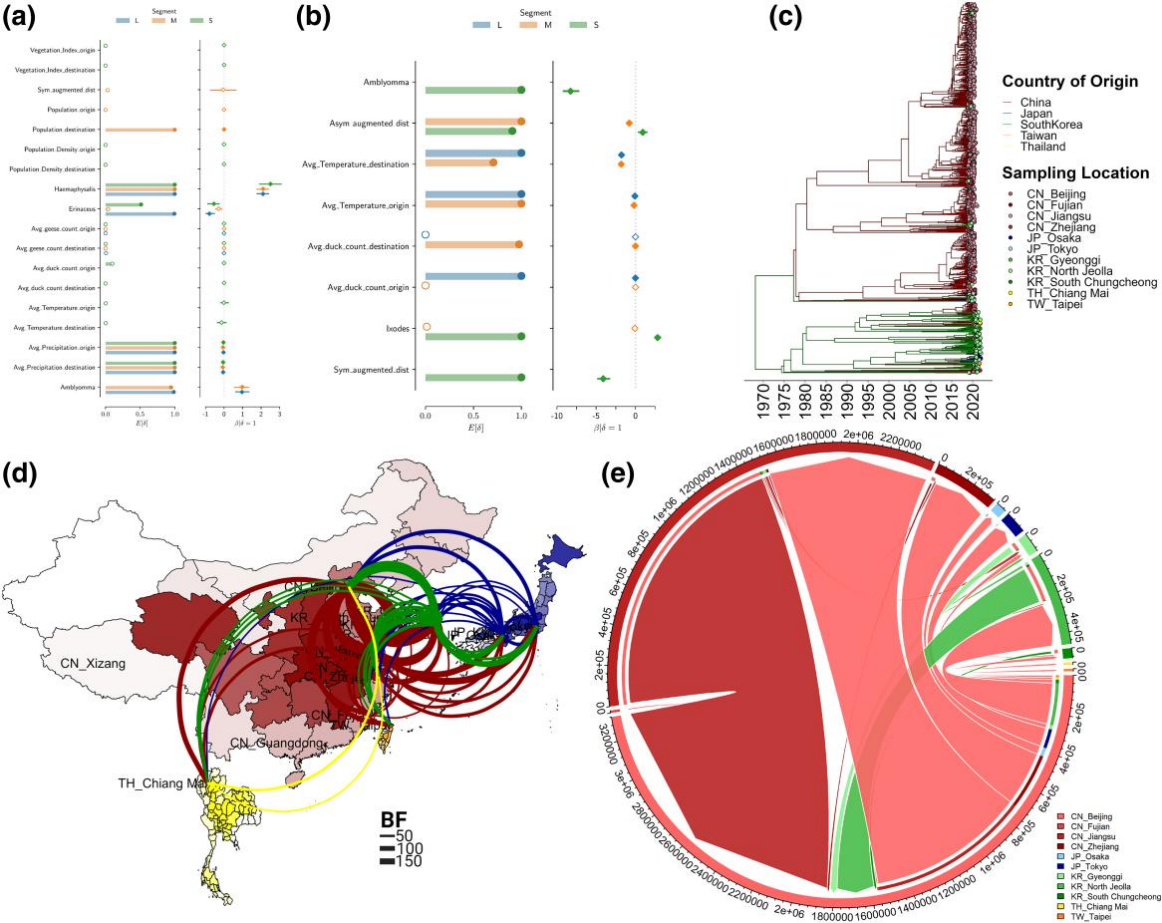
Detailed GLM phylogeographic analysis of SFTSV spread and influencing factors. a and b) GLMs uncover the relationships between SFTSV dispersal trajectory and different predictors, including environmental, ecological, and demographic factors (supplementary table S2, Supplementary Material online). The influence of each predictor on the virus spread was quantified by coefficient values and levels of significance. c) An MCC tree, identifying South Korea as the likely origin of the SFTSV proliferation. The analysis also identifies two predominant lineages: L1, with a significant presence in China, indicated by red, and L2, with significant dissemination in neighboring countries of Asia, denoted by green. d) Dynamic pathways of SFTSV of geographical movement denoted by Markov jump mappings. Only those transitions supported by a BF >20 are denoted. e) Transmission network of SFTSV summarized by the Markov jump event represented in a circular layout using the *circlize* package in R. This visualization facilitated the frequency and routes of virus importation, exportation, and intracountry dispersal. For inclusion probabilities and coefficients for all predictors, we refer to supplementary evaluation of predictors, Supplementary Material online.

To develop this framework further, we focused on the factors potentially influencing the dissemination of SFTSV in countries reporting the virus. Using a phylogeographic generalized linear model (GLM; Beard et al. 2014) we assessed 12 predictors spanning geographic, demographic, climatic, and ecological categories (see supplementary table S2, Supplementary Material online and Fig. 4a). Several predictors exhibited correlations exceeding 80% (see supplementary fig. S8, Supplementary Material online), so to delineate the impact of each on transmission, individual runs of the GLM were performed. We focused on predictors that were consistently influential across all three viral segments and enhanced our understanding of the dissemination process following the triple reassortment event that led to current-day SFTSV (Fig. 4a). Factors such as duck/geese migration dynamics, temperature, and vegetation did not align with trajectories and were rejected from the model. The predictors consistently supported for inclusion across all three SFTSV segments were the occurrence of *Haemaphysalis* species, the climate factor (precipitation: in both origin and destination countries), and the presence of *A. testudinarium* (Fig. 4a). The inclusion coefficient for the *Haemaphysalis* species showed a positive impact on the GLM diffusion, indicating the direction of the high viral transmission rate is toward the locations with a high occurrence of these species. In contrast, for *A. testudinarium*, a positive impact was only noted for the L and M segments, while a negative impact was observed for the S segment. These results bolster our earlier hypotheses regarding reassortment and vector jump events wherein the initial combination of L and M segments took place in *A. testudinarium* species. In contrast, the subsequent reassortment event between the M|L and S segments, which seemingly facilitated SFTSV's transmission to humans, appears to have been influenced or enabled by *H. hystricis*.

Discrete phylogeographic reconstructions displayed congruent topologies for all three SFTSV segments (Fig. 4c; see also supplementary fig. S9, Supplementary Material online). Interestingly, the root for each tree was traced back to South Korea, which contrasts with the prevailing consensus that SFTSV originated in China (Yu et al. 2011; Miao et al. 2020; Dai et al. 2022). Two major *lineages* are evident: one that proliferated within China (Lineage 1) and another that dispersed from South Korea (Lineage 2) (Fig. 4c; and supplementary fig. S9, Supplementary Material online). Analysis of mapped Markov jumps with a Bayes factor (BF) ≥50 highlighted significant intercountry transmission pathways. The primary routes emanated from South Korea, connecting to other Asian countries, notably Japan, Thailand, and China. Within China, however, the majority of Markov jumps illustrated a pattern of intracountry diffusion (Fig. 4d; and supplementary fig. S9, Supplementary Material online).

We summarized the intricate transmission dynamics across geographic locations using the Markov jump rewards in a circular plot, as depicted in Fig. 4 and supplementary fig. S9, Supplementary Material online. In this representation, the strains' origins are denoted by the inner circles, while the corresponding destinations are captured at the external arcs. To further delineate the directionality of movement, arrows connect origins to their designated destinations. A clear intercountry transmission dynamic emerges from the visual data. South Korea stands out as a primary source, introducing the virus predominantly to China. From there, the strains originating from South Korea spread further to other Asian nations, including Japan, Taiwan Province, and Thailand. Within the context of China, distinct intracountry diffusion patterns are evident. Notably, there is a marked dispersal of the virus originating from the Jiangsu region, primarily targeting Fujian region and subsequently reaching Zhejiang. Additionally, the Fujian region emerges as a central nexus, receiving a significant number of sequences from South Korea, underscoring its role in facilitating the SFTSV spread within China, as further detailed in Fig. 4e and supplementary fig. S9, Supplementary Material online.

### Unraveling Local Dissemination Dynamics of SFTSV Spread: Climate Variables as Modulating Factors

**Fig. 5.**
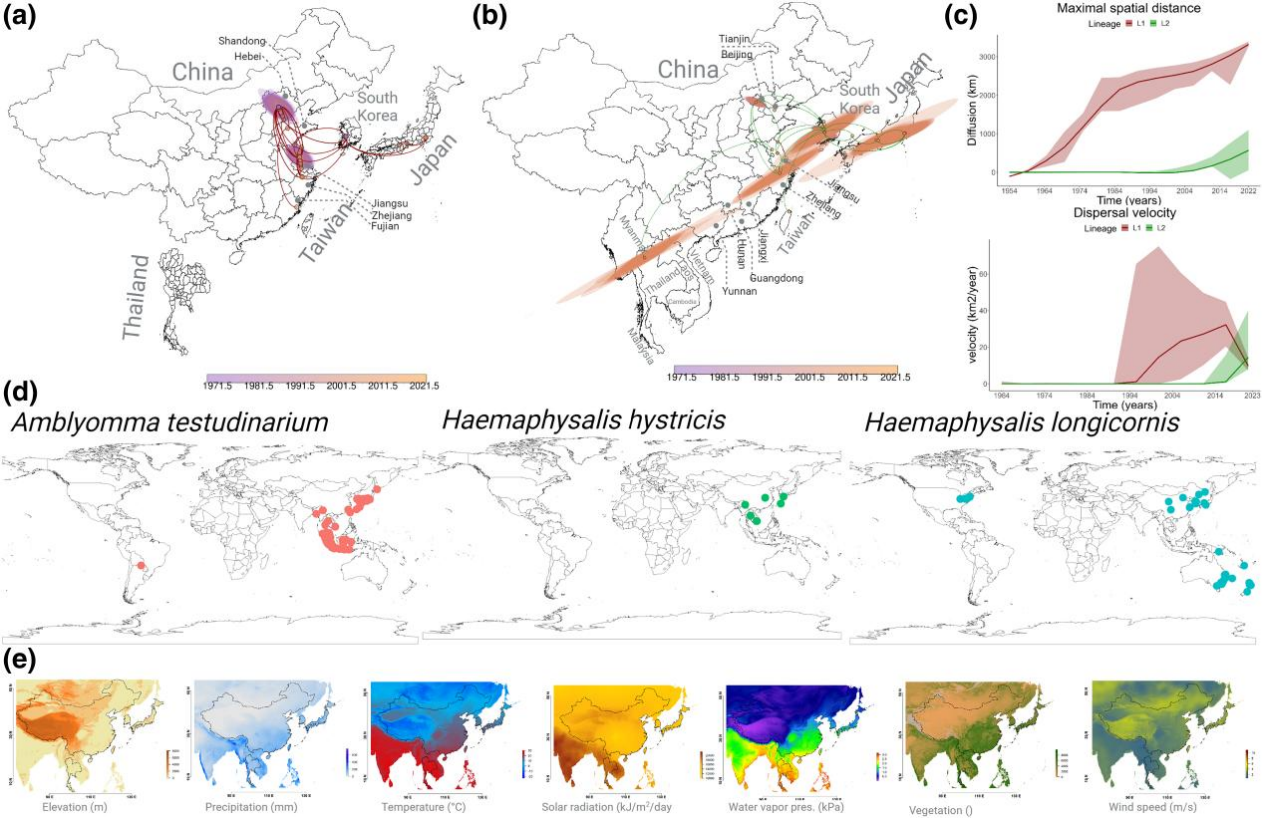
Tracing the historical dispersal and landscape dynamics of SFTSV lineages in Eastern Asia. Dispersal history reconstructions for SFTSV lineages a) L1 and b) L2, as identified in continuous phylogeographic analysis. Branch colors on the MCC trees correspond to curves in the spatial phylogeography, nodes and associated 80% HPD regions are time colored (see scale bars). c) Metrics of maximal spatial distance and dispersal velocity for each lineage (denoted in the plots) are presented, including weighted dispersal velocities and diffusion coefficients, with 95% HPD intervals. d) Global distribution of the primary tick vectors identified in this study (*A. testudinarium*, *H. hystricis*, and *H. longicornis*) as drivers of SFTSV emergence and spread. e) Impact assessment of environmental variables on lineage dispersal characteristics, such as resistance, conductance, attraction, and repulsion. Detailed statistical analyses are available in supplementary tables S4 to S7, Supplementary Material online.

Historical dispersal patterns of Lineages 1 (China) and 2 (South Korea) analyzed by continuous phylogeographic reconstruction suggest the critical role of strains from South Korea as the primary emergence point and the nexus for the initial propagation of Lineage L1 during the late 1970s to early 1980s (Fig. 5a). During this period, SFTSV appeared restricted to the Hebei and Jiangsu provinces of China (localized outbreaks represented as dense polygons; Fig. 5a). Following this initial phase, virus spread increased during the late 1990s and early 2000s targeting the Zhejiang, Shandong, and Fujian regions. This heightened transmission pattern could be attributed to the virus being locally transmitted throughout the eastern coast of China or via additional introductions from South Korea. Additionally, we identified a transmission chain to Japan that originated from South Korea from 2011 to 2020. While Lineage 2 appears to also have its origins in South Korea, its emergence is notably later, around the early 2000s. Swift local proliferation proceeded southward along China’s eastern seaboard, encompassing Zhejiang, Jiangxi, Yunnan, and other provinces, as well as the Beijing region (Fig. 5b). Outside of China from 2011 to 2022, Lineage L2 extended by local diffusion across South Korea, Japan, and Thailand (Fig. 5b). The extensive dispersion of SFTSV within Thailand suggests active transmission is occurring in neighboring countries like Viet Nam and Myanmar, who have reported instances of SFTSV but no sequences, and Laos, which has yet to report any cases (Fig. 5b). The dynamic transmission of L2 shows a unique spatiotemporal pattern, highlighting the regular exchange incidents between China, South Korea, and Japan with the more recent emergence of Taiwan and Thailand strains providing an additional footprint of the dissemination of SFTSV in Asia.

The post hoc evaluation revealed that Lineages L1 and L2 have different weighted diffusion coefficients and weighted branch dispersal velocities (supplementary table S3, Supplementary Material online) and the maximal spatial distances (Fig. 5c, top panel) exhibited distinct patterns in their spread over time. Lineage 1 steadily diffused starting around 1970 and significantly increased after 1994, reaching over 2,500 km by 2022. In contrast, the spatial diffusion of Lineage L2 remained static from its emergence until the early 2000s, but from 2005 onward, there was a marked expansion covering ∼500 km by 2022. Thus, L1 has a broader and longstanding spatial distribution, while L2 spread has been rapid over the past decade. Dispersal velocity for both lineages was near zero until the late 1980s, (Fig. 5c, top panel); however, this changed in the early 1990s when Lineage L1 surged and continuously increased until reaching its peak in 2014. Despite the ensuing sharp decline, L1 dispersal velocities remained notably higher than its initial levels, indicating active circulation in the region (Fig. 5c, top panel). The dispersal velocity of Lineage L2 only became detectable around 2010, but by 2014, a marked increase in its spread velocity allowed it to exceed L1 by 2020.

We hypothesize that the emergence trend of L2 may be the result of its increased adaptability to the new vector, *H. longicornis*, whose rise in occurrence began around 2000 and increased profoundly by 2010 (see Fig. 3). We plotted the geographic distribution of various tick species identified as key contributors to the emergence and dispersal of SFTSV (Fig. 5d) in conjunction with their temporal occurrences and the transmission dynamics of both SFTSV geographic lineages. Overlapping trends suggest that *H. hystricis* influenced the dispersal of L1, with its peak occurrence from 1990 to 1995 aligning with the increase in L1's dispersal velocity (Fig. 5a, c, and d). Moreover, its decline in population around 2010 corresponds with the decrease in L1's dispersal velocity in 2014. The synchronized rise in L2's dispersal velocity with the increasing prevalence of *H. longicornis* supports the hypothesis that this lineage is better adapted to this tick species vector. The recent spatial distribution of *H. longicornis* warns of the potential for the introduction of SFTSV into broader Asian regions, Australia, Indonesia, and North America.

Applying our spatially explicit phylogeographic model, we next explored the influence of several environmental variables (Fig. 5e) on the trajectory and dispersal velocity of SFTSV Lineages L1 and L2. Our analysis showed a statistically significant relationship between the dispersal trajectory of Lineage L1 and multiple climate variables, including humidity (measured by vapor water), precipitation, and temperature, when evaluated as attraction factors (BF >20; supplementary table S4, Supplementary Material online). None of these environmental variables acted as repulsion factors for L1 (supplementary table S4, Supplementary Material online). Similarly, for L2, these variables did not exhibit statistical significance when considered as repulsion factors; only wind speed was an attracting influence that impacted the dispersal direction of this lineage (supplementary table S4, Supplementary Material online). Utilizing univariate linear regression coefficients, we measured the role of environmental factors for dispersal velocity and duration. Simultaneously, we calculated *Q*-values to discern the proportion of variation in dispersal duration attributable to environmental variables, as opposed to geographical distance alone. Lineage L1's dispersal velocity appears to be impacted by a range of environmental variables: humidity, precipitation, solar radiation, temperature, vegetation, and wind speed, specifically when these variables were considered as conductance factors (supplementary table S5, Supplementary Material online). However, upon implementing a randomization analysis (supplementary table S6, Supplementary Material online) and considering those with a BF >20 in at least 2/3 rescaling analyses, only vegetation and solar radiation consistently impacted L1 dispersal velocity (supplementary table S7, Supplementary Material online).

These findings are confounding because they show that environmental variables appear to favor both the trajectory and speed of dispersal for Lineage L1, even though it has recently seen a decline in prevalence (Fig. 5c). Attempting to reconcile this disagreement, we revisited our initial hypothesis, wherein we believed the dispersal behavior of L1 strongly correlated with the prevalence of *H. hystricis* and the increasing prevalence of L2 aligned with the adaptive characteristics and spreading tendencies of *H. longicornis*. We thus considered the unique biological traits of each tick species, along with other external factors like competition and environmental conditions. *H. hystricis* tends to be host-specific, primarily targeting mammals and showing a preference for warm and humid environments, whereas *H. longicornis* exhibits a more adaptable nature, with a wide host range encompassing mammals, migratory birds, and reptiles. The latter's versatility extends to its environmental adaptability, as tick species can be found in diverse settings, from rural landscapes to urban locales, and hence considered invasive (Tufts et al. 2021). A crucial element that emerged from our study was the (supplementary fig. S10a, Supplementary Material online) exponential growth in pesticide application for agricultural purposes in China between 1990 and 2010. Our analysis revealed a pronounced negative correlation (−1.00) between the use of pesticides (supplementary fig. S10b, Supplementary Material online) and the prevalence of *H. hystricis*, in contrast to a modest positive correlation (0.3) with *H. longicornis* (supplementary fig. S10c, Supplementary Material online). These results indicate that the surge in pesticide applications across China has detrimentally affected the range and population of *H. hystricis*, without noticeably impacting *H. longicornis*. Thus, pesticides may be functioning as a selective pressure, contributing to the expansion of *H. longicornis* at the expense of *H. hystricis*, to explain the recent shift from L1 to L2 as the more prevalent lineage over the last decade.

## Discussion

In the present study, we have described the genetic diversity and history of SFTSV, unraveling the complex mechanisms and ecological factors that have shaped its evolutionary trajectory and geographic expansion. The refined clade structure uncovered here challenges previous classifications and emphasizes a clade demarcation more closely correlated with geographical distribution (Fig. 1). In other words, our analysis is not consistent with previous assignments of genotypes for SFTSV (Li et al. 2021). This pattern highlights the critical influence of geographical factors on viral evolution and spread, pointing out the role of local adaptation in its diversification. Contrary to previous studies, we did not find reassortants of SFTSV. The use of ultrametric trees to accurately detect reassortment events has clarified prior misinterpretations (Li et al. 2021; Dai et al. 2022; Wang et al. 2023) and emphasized the necessity of accounting for the evolutionary time of the virus (de Vienne 2019; Fig. 2). Our detection of true recombination events was also reduced in frequency when demanding parental strains to coincide in time and space. Nevertheless, the contribution of recombination alongside the identification of distinct selective pressures acting on various viral segments, presents a description of the actual mechanisms driving SFTSV diversity. Differences between genomic segments suggest a balancing act with each evolving under varied evolutionary forces. The L segment, for instance, seems to be subjected to intense purifying selection, while M and S may be influenced by relaxed or even positive selection (Fig. 2). This differential selection reflects a virus adjusting its genetic makeup to optimize survival and transmission in a changing environment.

Temporal and spatial analyses yielded insights into SFTSV's origin and the factors influencing its dispersal. The use of molecular clock dating has enabled us to estimate the MRCA for each viral segment, revealing a staggered timeline of emergence (Fig. 3). The earlier MRCA of the M segment compared with the L and S segments suggests a complex temporal evolution for SFTSV, with an extended evolutionary history for M. Our population dynamics analysis indicates a notable gap between the MRCA and the increase in genetic diversity observed from the 1970s onward (Fig. 3). We propose two hypotheses to explain this behavior: (i) *evolutionary stasis*: the M segment experienced a prolonged period of minimal evolution, possibly attributed to an unchanging ecological niche where neither the virus nor its primary vectors and hosts underwent significant shifts and balanced co-evolution favored genomic stability; (ii) *undetected reassortment*: the tMRCA in 1270 might represent an ancestral lineage of the M segment that later became rare or extinct. Post-1970 dynamics could reflect a more recent, and more adaptable M lineage that emerged due to significant reassortment events. Nevertheless, the concurrent increase in genetic diversity for both the L and M segments around 1970 suggests a shared, significant evolutionary event like reassortment that may have facilitated the adaptation and spread of the virus. The S segment emergence, dated around 1985, aligns with retrospective evidence of SFTSV presence in specimens dating back to 2005 (Liu et al. 2012). The subsequent 20-year interval marks a critical period for SFTSV that further shaped its evolutionary trajectory. Our findings highlight a strong temporal correlation between the genetic evolution of SFTSV and the prevalence of different tick species (Fig. 3). The rise and fall of tick populations, particularly *A. testudinarium* and *H. hystricis*, appear to coincide with key evolutionary milestones of the virus, suggesting their potential role in the transmission and genetic rearrangement of SFTSV. The recent increase in genetic diversity from 2010 to 2022 could reflect SFTSV adaptation to *H. longicornis*, as evidenced by the increasing incidence of SFTSV in different geographical regions. The discrete phylogeographic analysis provides new perspectives on the origin and movement patterns of SFTSV, pointing to South Korea as the likely source, challenging the prevailing hypothesis of a Chinese emergence (Yu et al. 2011; Li et al. 2021; Dai et al. 2022) and reshaping our understanding of its progression through Asia (Takahashi et al. 2013; Tran et al. 2019; Win et al. 2020; Rattanakomol et al. 2022). Two dominant lineages (L1 and L2) have further diversified along distinct geographical and temporal lines (Fig. 4), with South Korea acting as the epicenter for spread of SFTSV to other countries including Japan and China (Fig. 4). This information is critical as it implies the need to reassess and potentially revise surveillance and containment strategies, considering South Korea's significant role in SFTSV propagation. The continuous phylogeographic analyses indicate that Lineage L1 remained localized within certain provinces of China until the late 1990s before it expanded into adjacent areas. In addition, we demonstrate a correlation between the historical, localized spread of Lineage L1 in China and the prevalence of *H. hystricis*, implicating this species as the primary vector in the early spread of the virus (Figs. 3 and 5). The dispersal of Lineage L2, emerging much later and exhibiting a swift southward spread down the east coast of China, features a different evolutionary trajectory. Its rapid local proliferation and expansion, coinciding with the rise of *H. longicornis*, suggested a shift in vector ecology (Figs. 3 and 5) that augmented the broad dissemination of the virus. The dispersion velocities of the two lineages add an additional layer to our understanding. While Lineage L1 has exhibited a continuous spread with a recent decline in velocity, Lineage L2 has shown a stark acceleration in recent years, surpassing the dispersal velocity of L1. This observation may reflect an increased adaptability of L2 to environmental changes or ecological pressures, such as those exerted by the pervasive use of pesticides in agricultural practices.

Despite the declining prevalence of Lineage L1, environmental conditions like humidity, precipitation, and temperature significantly influence its dispersal trajectory and velocity, therefore highlighting the potential for climate change to further modify the spread of SFTSV. In contrast, Lineage L2, with its rising prevalence, appears to be exploiting new ecological niches facilitated by the adaptability of *H. longicornis*, an invasive species that feeds promiscuously on a variety of hosts, with migratory birds being of greatest concern. Indeed, the diversity of animal hosts from which Lineage L2 sequences (Clades II and III; Fig. 1) originate illustrates this point and the danger that host-jumps pose for increased transmission to humans. This situation is further exacerbated by anthropogenic factors, such as the use of pesticides, which have added another selective pressure on the tick population, inadvertently influencing the evolutionary dynamics of SFTSV.

Our findings provide a clearer picture of the historical spread and current dynamics of SFTSV, as well as the complex interplay of ecological, environmental, and anthropogenic factors that have shaped its current trajectory. The potential for SFTSV to exploit new vectors and spread to novel regions should prompt a reevaluation of vector control strategies and public health policies, particularly in areas like South Korea where both lineages have emerged and acted as a source for the introduction of the virus to different locations. The recent presence of SFTSV in Southeast Asia and its demonstrated adaptability to *H. longicornis* warrant increased surveillance and research to prevent its establishment in broader geographical areas, including its introduction into Australia, Indonesia, and North America. Recognizing the current elevated circulation of *H. longicornis* in these areas and this possibility, Miao et al. (2020) have estimated the likelihood of SFTSV introduction. The insights gained from our study should therefore inform future strategies to mitigate the spread of SFTSV and underscore the need for an integrated approach to disease surveillance that encompasses virology, vector ecology, and climate science. As we consider other vector-borne pathogens more generally, we must also endeavor to integrate these evolutionary considerations into predictive models that account for the role of environmental and anthropogenic factors in their spread.

## Materials and Methods

### Genomic Data Collection and Data Curation

All SFTSV sequences utilized in this study were retrieved from the GenBank database on 2023 March 1. Our initial dataset involved both partial and complete-genome sequences across all three genomic segments (supplementary data tables S1 to S3, Supplementary Material online available in the repository: https://github.com/LesterJP/SFTSV_Research). A deduplication analysis was conducted following the methodology described by Perez et al. (2021). To attach these sequences with relevant metadata, we extracted additional details such as the year of collection, country of origin, strain name, and host species. Metadata extraction was accomplished using the *gbmunge* script, available at GitHub: https://github.com/sdwfrost/gbmunge, supplemented by a custom R script. This latter script was specifically designed to concatenate information for those strains for which all three genomic segments were available (supplementary data table S4, Supplementary Material online, available in the repository: https://github.com/LesterJP/SFTSV_Research).

### Sequence Alignment and Phylogenetic Inference

All downloaded sequences, covering the three genomic segments of SFTSV, were aligned using the MAFFT software v7.453 (Rozewicki et al. 2019) with settings for a *localpair* option alignment. The alignments were then utilized for ML phylogenetic inference using IQ-TREE2 (Nguyen et al. 2015). Initially, the *ModelFinder* algorithm was employed to identify the most appropriate nucleotide substitution model based on the Bayesian Information Criterion. Subsequently, ML phylogenetic trees were constructed. To evaluate the reliability of the branching patterns within the ML trees, we conducted 10,000 replicates each of the Shimodaira–Hasegawa Approximate Likelihood Ratio Test and Ultrafast Bootstrapping.

### Genotype and Reassortment Evaluation

To identify genotypes within the SFTSV species, we modified the approach originally detailed in Perez et al. (2021). Specifically, we used PASC implemented in the Sequence Demarcation Tool (SDT; Muhire et al. 2014) for taxonomic classification. Briefly, all unique, complete-genome sequences for all three segments of SFTSV in our dataset were used as input into the SDT software, which utilizes a robust Needleman–Wunsch algorithm for pairwise sequence alignments. This software calculates sequence identity, excluding indel positions. Moreover, we plotted the PASC distributions for all three genomic segments and determined the cutoff values as previously described in Perez et al. (2021, 2023). Briefly, based on the taxonomical organization used (in this case, species level), the external cutoff (valley in the distribution) will indicate the cutoff of species level, and subsequent cutoff (internal valleys) will indicate genotypes and subgenotypes, respectively (Citations here all Perez analysis PASC). Subsequently, we examined the formation of statistically supported clades, using these PASC-determined genetic distance values as cutoffs and a bootstrap support threshold of 70% in the software cluster picker (Ragonnet-Cronin et al. 2013).

To determine the potential presence of reassortant strains in SFTSV, we deployed a computational workflow as follows: initially, primary sequence datasets for the L, M, and S viral segments were curated to include only strains with complete genomes, which were then merged based on strain names. These consolidated datasets were used for inferring three ML phylogenetic trees, as detailed in the Sequence Alignment and Phylogenetic Inference section. Each ML tree was rooted at the most distant node, maximizing the sum of all patristic distances, and then converted to ultrametric trees by using the *ape* R package. This crucial step was implemented to account for those strains that could be circulating simultaneously within the same host, a prerequisite for the occurrence of reassortment events. After ultrametrization, the trees were binarized using the *multi2di* function (also from the *ape* R package) and transformed into dendrograms. We employed the *untangle* and *tanglegram* functions from the *dendextend* R package to generate tanglegrams, offering a visual comparison of segment-specific tree topologies. We further quantified the topological differences between the pair of trees by computing the RF distances between the trees, utilizing the *RF.dist* function from the *ape* R package. In a subsequent stage of our analysis, we carefully inspected the potential reassortant strains initially labeled through tanglegram visualization, by establishing a threshold for cophenetic distance differences that was aligned with the previous PASC threshold. Thus, cophenetic distances for each ultrametric tree were calculated using the *cophenetic* function from the *ape* R package. To visualize the distribution of these differences, histograms and heat maps were created. These graphical representations provide a comprehensive, multidimensional view of cophenetic discrepancies among potential reassortant strains.

### Homologous Recombination and Adaptive Selection

To determine the presence of homologous recombination in SFTSV sequences, we employed the methodology recently described in Perez et al. (2023b). We initially screened for recombination hot spots in the complete-genome sequences of the L, M, and S segments using the Recombination Detection Program (RDP5; Martin et al. 2020). This involved the application of six specific algorithms: RDP, GENECONV, Bootscan, MaxChi, Chimera, SisScan, and 3SEQ, with parameter settings adjusted according to the recommendations in the RDP5 manual. Sequences were considered recombinant when detected by at least four of the six algorithms and yielding statistical support with a *P*-value of 0.01 by applying Bonferroni's correction for multiple comparisons. To further ensure the accuracy of our findings and minimize the risk of false positives, we confirmed recombination results by four different approaches. This included (i) cross-validation with the Pairwise Homoplasy Index test, (ii) confirming high bootstrap values exceeding 75% in BootScan analysis, (iii) validating phylogenetic branch divisions using the Shimodaira–Hasegawa test, and (iv) we discarded false positive recombinants if their parental strains did not emerge from a closely related ancestor (defined as fewer than 10 branches from the node) and did not circulate temporally within ∼2.4 year, considered as the uncertainty yielded by the median 95% HPD of the most external nodes of MCC trees for the three genomic segments.

To assess adaptive selection in the coding regions of all three SFTSV segments, we employed a two-step process (Pérez et al. 2011): we calculated the amino acid sequence entropy using the DAMBE software (Xia and Xie 2001), and the difference between nonsynonymous and synonymous substitution rates (d*N*/d*S*) was computed using the SNAP web utility based on the Nei and Gojobori method (Nei and Gojobori 1986). These metrics were then plotted against each other to examine the evolutionary pressures on the SFTSV coding regions.

### Temporal and Demographic Bayesian Inference

From our initial set of sequences (see supplementary data tables S1 to S3, Supplementary Material online, available in the repository: https://github.com/LesterJP/SFTSV_Research), we focused on those that had associated isolation dates. Initially, this curated dataset was analyzed using *TreeTime* (Sagulenko et al. 2018) to identify and remove any statistical outliers (see supplementary fig. S4, Supplementary Material online). To examine the existence of a molecular clock in the dataset, we employed two key strategies to evaluate the temporal signal (Perez et al. 2023): (i) Root-to-tip regression analysis (Rambaut et al. 2016), and (ii) the BETS test (Duchene et al. 2020). The root-to-tip distance was calculated using TempEst (Rambaut et al. 2016), employing heuristic residual mean squared methods. As input, we used ML trees that were reconstructed with IQTREE2 from datasets where outliers had been previously removed. The BETS test was performed using BEAST v.1.10.4 (Suchard et al. 2018) to statistically evaluate two different models: heterochronous and isochronous. These models were compared using log marginal-likelihood estimators (log MLEs), specifically path sampling and stepping-stone sampling methods (Baele et al. 2012). Two molecular clock approaches were assessed: a strict clock and an uncorrelated relaxed log-normal clock. Prior distributions were set with an exponential mean of 1.0 for the clock and an exponential population size with a mean of 10.0 and an offset of 0.5 for the coalescent prior. Analyses were executed with a chain length of 9 × 10^8^ interactions, from which the initial 10% was discarded as burn-in. For each genomic segment, four separate runs were conducted to account for each hypothesis and clock model. MLE was obtained using path sampling and stepping-stone sampling, with a chain length of 9 × 10^6^ and 900 path steps, utilizing the default beta distribution for the path step.

Time-scaled phylogenies for all the three genomic segments of SFTSV were generated using BEAST 1.10.5, with the BEAGLE 3 library (Ayres et al. 2019) employed to enhance computational efficiency. The substitution process was set based on a General Time Reversible model with Gamma distribution (GTR+Γ). Based on the results from the BETS analysis, a relaxed molecular clock model with an underlying log-normal distribution was utilized for estimating branch-specific evolutionary rates. The SkyGrid tree prior model (Hill and Baele 2019) was selected for the analysis. For each genomic segment, eight independent runs were executed, each sampling from the Markov Chain Monte Carlo (MCMC) chains at intervals of 9 × 10^8^ generations with sampling every 9 × 10^5^ generations. Subsequent evaluation of convergence was determined by the effective sample size (ESS) parameter estimates, with values of ESS >200, determined by using the software Tracerv1.7.

### Discrete Phylogeographic Reconstruction

We initially conducted separate discrete phylogeographic reconstructions for all three segments of SFTSV sequences. The analysis was performed using BEAST 1.10.4 (Suchard et al. 2018) with a discrete trait diffusion model (Lemey et al. 2009), with the computational efficiency of these estimations enhanced by the BEAGLE 3 library (Ayres et al. 2019). The analyses utilized the HKY substitution model and employed a relaxed clock model with rates sourced from a log-normal distribution. Additionally, a SkyGrid coalescent model (Hill and Baele 2019) was chosen as the phylogenetic tree prior. Furthermore, we adopted the GLM extension of the discrete diffusion model (Beard et al. 2014) to simultaneously evaluate the migration patterns of lineages across specific locations and to explore the possible influence of external factors on transition rates between these locations. This approach enables the study of how external variables could affect the migration rate of SFTSV lineages among identified locations. The strength of the association for each examined predictor was quantified using GLM coefficients, with statistical support estimated through the BF (Kass and Raftery 1995). Within this study, we considered the region of isolation as discrete states and tested a total of 12 predictors summarized in supplementary table S1, Supplementary Material online. Each predictor was obtained as follows:

- *sym augmented dist*: We began by defining geographic coordinates for specific locations across multiple countries and regions of associated to the metadata of the SFTSV sequences. For each pair of selected locations, the Haversine distance was then calculated, since this distance metric considers the curvature of the Earth. Each location was assigned a weight based on its proximity to other locations. The weight was calculated as the number of other locations within a 200 km radius, serving as an indicator of geographic influence. The distance between Locations *i* and *j* was modified by an average factor that considers the weights of both Locations *i* and *j* under the formula (1).
  (1) $$\left(1+\frac{\mathrm{Weight}\;\mathrm{of}\;i+\mathrm{Weight}\;\mathrm{of}\;j\;}{2}\right)\times \mathrm{Haversine}\;\mathrm{distance}\;\mathrm{between}\;i\;\mathrm{and}\;j$$
- *asym augmented dist*: The same condition as for *sym augmented dist* was applied but the factor that considers the weight of locations was modified, see formula (2)
  (2) (1+Weightofi)×Haversinedistancebetweeniandj
- *Population density*: Population densities for the locations in the study were obtained from the Open access spatial demographic datasets built using transparent approaches. https://hub.worldpop.org/ accessed on 2023 April 12. We used the unconstrained individual countries 2000 to 2020 UN adjusted raster at 1k resolution.
- *Precipitation*: The precipitation data were downloaded from a NetCDF file that provides monthly precipitation data from 2011 to 2020 at a 0.25° spatial resolution available at https://climatedataguide.ucar.edu/. Finally, the precipitation data were averaged for each location of the study.
- *Temperature*: Temperature data were retrieved from the NASA POWER (Prediction of Worldwide Energy Resources) database. Specifically, the data represented daily surface air temperature (T2M) from 2010 January 1 to 2022 December 31, at https://power.larc.nasa.gov/. A custom R function was employed to fetch and process temperature data for each specified location. Finally, the temperature data were averaged for each location of the study.
- *Vegetation*: Normalized Difference Vegetation Index-3rd generation (NDVI) using the Global Inventory Monitoring and Modeling System available at https://climatedataguide.ucar.edu/, was used to create a raster file of Vegetation for the Asia region. Then the vegetation index value was extracted for each valid spatial point from the raster file.
- *Ixodes*: Tick occurrence data for the species *Ixodes persulcatus* were gathered using the GBIF API with the rgbif package in R. The data were filtered to include occurrences from China, Japan, South Korea, Thailand, and Taiwan with precise geographic coordinates. We also accounted for a proximity measurement in a way that for each location of interest, the nearest occurrence data were identified using the Vincenty distance formula as implemented in the *geosphere* package in R. A pairwise count matrix was computed to measure the association between tick occurrences across different provinces. Finally, the matrix was normalized to account for the influence of distance.
- *Amblyomma*: The same approach used for the predictor *Ixodes* was applied to occurrence data for the species *A. testudinarium*.
- *Haemaphysalis*: This predictor was created similar to the *Ixodes* predictor, but in this case the occurrence for the *Haemaphysalis megapinosa*, *H. hystricis*, *Haemaphysalis formosensis*, *H*. *flava*, and *H. longicornis* were combined into a single predictor.
- *Erianceus*: Considering the study from Zhao et al. (2022), who reported Hedgehogs (*Erinaceus amurensis*) as an amplifying host of SFTSV we included this predictor and applied the same approach as the predictor *Ixodes* from the occurrence of the *E. amurensis* in the different locations of the study, the resulting matrix was also normalized to account for the influence of distance for this species.
- *Geese movement:* In light of confirmed detections of SFTSV in geese, substantiated by available genetic sequences for this host species, we incorporated the dynamics of duck migration as a critical predictive variable in our study. We initially accessed the eBird Database on 2023 April 21 https://science.ebird.org/. Utilizing a custom R script, we extracted essential data points such as the occurrence, latitude, longitude, and date of each individual goose sighting. To ensure the relevance of this data to our predefined key locations, we filtered the information to only include sightings within a 2.25° radius around the latitude and longitude coordinates of these key points, which were considered the locations in the discrete traits in our phylogeographic analysis. For each location, we then aggregated the goose occurrence data by month. Given the high degree of correlation found across various months, we simplified this predictor by calculating an average goose count per location.
- *Ducks movement:* Building on our methodology for assessing duck migration dynamics, we also incorporated duck migration patterns as another predictive variable. Similar to the duck analysis, we extracted geese sighting data from the eBird Database, focusing on our predefined key locations. We used the same R script for data extraction and filtering. We also aggregated the monthly occurrences and as with ducks, we found a high correlation across months, leading us to simplify the predictor by using the average duck count per location.

We estimated all Markov jump counts to the trees by selecting *Reconstruct complete change history on tree*, that allows to estimate both the frequency and the timing of each jump or state transition. Eight independent MCMC runs were executed, each analysis was set for 9 × 10^8^ iterations and sampling at intervals of 8 × 10^5^ iterations. To ensure robustness, the posterior distributions from these runs were combined after excluding the initial 10% of sampled trees from each chain. The convergence and mixing properties of the chains were evaluated using Tracer v1.7. The ESS values >200 confirmed the adequacy of the sampling and the reliability of our estimates. Subsequently, a MCC tree was generated using TreeAnnotator v1.10. We reported Markov jumps between discrete locations and considered statistically supported (BF) values >20 as described by Kass and Raftery (1995). A post hoc analysis was conducted to summarize the Markov Jumps estimates for transitions between discrete states, utilizing the *TreeMarkovJumpHistoryAnalyzer* tool. This tool is part of the BEAST codebase and was accessed on 2023 September 15, from its GitHub repository. For visualization of these transitions, we employed the R package *circlize* (Gu et al. 2014).

### Continuous Phylogeographic Analysis

To elucidate the local spread of the two main lineages identified in our discrete phylogeographic analyses, we partitioned the dataset and conducted separate Bayesian phylogenetic assessments using BEAST1.10.4 (Suchard et al. 2018). A relaxed clock model and a SkyGrid coalescent prior were implemented for this purpose. Our continuous phylogeographic analyses were performed using a relaxed random walk diffusion model (Lemey et al. 2010), using a gamma distribution to account for among-branch variability in diffusion rates. This approach allowed us to infer the geographical coordinates of internal nodes on the phylogenetic tree, mapping the evolutionary trajectory of each lineage from its origin to its current location. In alignment with our temporal and discrete phylogeographic analyses, we executed eight independent MCMC runs, each comprising 9 × 10^8^ generations and sampled at intervals of 9 × 10^5^ generations. Convergence and model fit were validated using Tracer 1.7, confirming that all ESS >200. An MCC tree was constructed using TreeAnnotator 1.10. To further analyze the spatiotemporal dynamics of the virus spread, we conducted post hoc analyses using the R package *Seraphim* (Dellicour et al. 2016a). This allowed us to extract spatiotemporal information embedded in the posterior tree distributions. Specifically, we calculated two key dispersal metrics: the weighted diffusion coefficient (*expression 3*) and the weighted branch dispersal velocity (*expression 4*). These metrics were computed based on the geographic distance (measured in kilometers) and time duration (measured in years) for each branch in the phylogenetic tree. These metrics provide a detailed understanding of the viral spatial and temporal diffusions (Dellicour et al. 2016a).


(3) $$D_{\mathrm{weighted}}=\frac{\sum_{i=1}^{n}\;d_{i}^{4}}{\sum_{i=1}^{n}\;t_{i}^{2}}$$

(4) $$v_{\mathrm{weighted}}=\frac{\sum_{i=1}^{n}\;d_{i}}{\sum_{i=1}^{n}\;t_{i}}$$

### Evaluation of Landscape Phylogeography

We employed landscape phylogeographic analyses (Dellicour 2016b) to examine the role of environmental factors on the spatial and temporal dynamics of SFTSV. Specifically, we used two analytical methods to assess the effects of environmental variables on both the coefficient for dispersal location (Dellicour et al. 2020) and dispersal velocity (Dellicour et al. 2020) of SFTSV. These methods involve comparing inferred spatially annotated phylogenetic trees with randomized versions of these trees. In the randomized trees, the geographic locations of phylogenetic branches are rearranged while maintaining the original time-scaled topology, branch length, branch duration, and root position (Dellicour et al. 2016a, 2016b, 2020). Initially, we investigated whether SFTSV lineages show attraction or repulsion to areas with certain environmental conditions. We extracted and averaged the environmental values at the trees nodes positions to obtain a posterior distribution of mean environmental values for each factor (see environmental variables). The BF (Kass and Raftery 1995) was used to compare the averaged values between inferred and randomized trees. In this context, pe was defined as the frequency at which the environmental values from inferred trees were either greater or smaller than those from randomized trees. For statistics support a BF >20 was considered strong evidence, and a value between 3 and 20 as positive evidence (Kass and Raftery 1995). Secondly, we explored the impact of environmental factors on the velocity of SFTSV lineage dispersal. We computed an “environmental distance” for each branch in both the inferred and randomized trees using the least-cost path model implemented in *Seraphim* R package (Dellicour et al. 2016a). These distances were estimated using two different types of rasters: one based on specific environmental variables and a null raster in which all cell values are set to “1.” This last one (null raster) serves as a negative control, a crucial step for mitigating the risk of false positives in the analysis, as point out by Dellicour et al. (2018). We further examined the impact of environmental factors on viral lineage dispersal velocity using the expression (5). Thus, we transformed the raster cell values of each environmental variable, where *v*_t_ and *v*_o_ are the transformed and original cell values, respectively, and *v*_max_ is the maximum cell value in the raster.


(5) $$v_{t}=1+\frac{kv_{o}}{v_{\max}}$$

This rescaling parameter *k* allowed us to test different strengths of conductance or resistance relative to a “null” raster where all cell values are set to “1.” Specifically, we generated three distinct rasters for each environmental variable using rescaling factors *k* of 10, 100, and 1,000. We then we utilized a statistic *Q*, to assess the influence of environmental variables on viral dispersal velocity, with $Q=R_{\mathrm{env}}^{2}-R_{\mathrm{null}}^{2}$, where $R_{\mathrm{env}}^{2}$ is the coefficient of determination obtained when regressing branch durations against environmental distances based on transformed environmental rasters, and $R_{\mathrm{null}}^{2}$ is the coefficient of determination when the same is done using a null raster (Dellicour et al. 2018). The significance of *Q* was examined across the 1,000 trees sampled from the posterior distribution and for each environmental raster transformation. An environmental factor was considered to have potential influence on dispersal velocity if both its distribution of regression coefficients and its *Q*-values were predominantly positive, specifically with >90% of the values exceeding zero. When this condition was satisfied, Bayesian support for the *Q* distribution was verified in contrasting it with a null distribution of *Q*-values, which were computed based on randomized phylogenetic branches. We then formalized the robustness of these findings using BF approximations, like the methodology we employed for evaluating the influence of environmental variables on the geographical distribution of SFTSV lineages. Importantly, an environmental factor was confirmed to significantly impact dispersal velocity, if it yielded a BF >20 in at least two of the raster transformations. For both landscape phylogeographic approaches we tested the following environmental variables (rasters) within an Asian extent region defined by the geographical coordinates from 70 E° to 150 E° longitude and 5 N° to 55 N° latitude, and the data were rasterized using the *raster* R package:

- *Elevation*: We initially utilized the *rnaturalearth* R package to obtain medium-scale world map data. This data were then filtered to include Asian defined region. The elevation data were then extracted using the *elevatr* R package by specifying a zoom level of 5 and WGS84 projection system.
- *Precipitation*: The data for this study were obtained from NetCDF files available on the Climate Data Guide website (https://climatedataguide.ucar.edu/). These files provide monthly precipitation data spanning from 2011 to 2020 and offer a 0.25° spatial resolution. The data were averaged for each location within our study area, which encompasses the Asian extent region.
- *Temperature*: The data for temperature were obtained as previously described for this predictor in the GLM evaluation. As an environmental variable, the temperature data were processed to create a raster stack and subsequently averaged to obtain an annual mean temperature raster for the region of interest.
- *Solar radiation*: The solar radiation data were obtained from the Copernicus Climate Data Store, specifically from the Coupled Model Intercomparison Project Phase 5 monthly single-level dataset on 2023 May 21 (https://cds.climate.copernicus.eu/). The variable of interest was Top of Atmosphere incident solar radiation. The dataset was cropped to focus on an area of interest. The annual average solar radiation was calculated from the monthly data to produce a raster layer.
- *Water vapor*: The water vapor data were obtained from NASA's Earth Observing System Data and Information System, available at the Global Science and Visualization website on 2023 May 21 (https://neo.gsfc.nasa.gov/view.php? datasetId=MYDAL2_M_SKY_WV). The raster file was directly downloaded from the website and cropped to focus on an area of interest.
- *Vegetation*: The vegetation data were collected from the NOAA STAR Vegetation Health Product (https://www.star.nesdis.noaa.gov/). We specifically focused on the NDVI, a widely used indicator that quantifies vegetation greenness and is calculated from the visible and near-infrared light reflected by vegetation. For the scope of this study, a raster file representing NDVI, specifically focusing on Greenness (no noise NDVI), was directly downloaded from the site. This raster was then cropped to match our area of interest.
- *Wind speed*: The wind speed data were sourced from the Global Wind Atlas (https://globalwindatlas.info/en), a comprehensive repository providing wind speed information at various altitudes. The dataset utilized in this study was collected at a 50 m resolution and focused on mean power density to neutralize the effects of wind directionality. After downloading, the raster data were cropped to our region of interest.

### Ecological Parameters Analyses

We started our analysis by examining the frequency distribution of various tick species that serve as hosts/vector for the SFTSV virus, utilizing metadata extracted from the sequence data. We further analyzed the temporal occurrence of the tick species that we identified associated with SFTSV. For this purpose, we used the *rgbif* R package to query the Global Biodiversity Information Facility (GBIF). The species examined included *H. megapinosa*, *H. hystricis*, *H. formosensis, H. flava*, *H. longicornis*, *A. testudinarium*, and *I. persulcatus*. We performed our search to a maximum of 20,000 records per species within the defined region of interest that included species names, collection dates, and coordinates. To elucidate trends in species occurrences over time, we employed locally estimated scatterplot smoothing (LOESS), a nonparametric technique that fits a smooth curve through data points in a scatter plot using local weighted regression. In this study, the LOESS model was applied with a span parameter of 0.8, signifying that each smoothed value was influenced by 80% of its neighboring data points. The smoothing process was executed using the *geom_smooth* function from the *ggplot2* R package. This methodology enabled us to visualize and interpret the annual fluctuations in occurrence counts for each tick species, without imposing rigid assumptions about the relationship between the year and the frequency of occurrences.

To investigate the potential impact of agricultural expansion in China on the populations of *H. hystricis* and *H. longicornis*, we incorporated data on annual pesticide usage obtained from the FAOSTAT database (https://www.fao.org/faostat/en/) accessed on 2023 October 10. We further clean this dataset to retain only variables essential for the study. Both the pesticide usage and tick occurrence datasets were then aggregated by year to align with the same temporal frame. We then estimated a *Pearson* correlation coefficient to examine the relationships between annual pesticide usage and the annual counts of tick occurrences for each species. This quantitative approach allowed us to assess the degree to which changes in pesticide usage are correlated with fluctuations in the occurrence of these two tick populations.

## Supplementary Material

msae173_Supplementary_Data

## Data Availability

All the raw data included in the current study are publicly available in the repository: https://github.com/LesterJP/SFTSV_Research.
