# Supplementary material for: Ecological Changes Exacerbating the Spread of Invasive Ticks has Driven the Dispersal of Severe Fever with Thrombocytopenia Syndrome Virus Throughout Southeast Asia
Source: Mol Biol Evol. 2024 Aug 28;41(8):msae173. doi: 10.1093/molbev/msae173 (PMC11349436; doi:10.1093/molbev/msae173)
Supplement: msae173_Supplementary_Data [file msae173_supplementary_data.pdf]

Supplementary Information

Ecological changes exacerbating the spread of invasive ticks has driven the dispersal of SFTS virus throughout southeast Asia

Lester J. Pérez[a,b], Guy Baele[c], Samuel L. Hong[c], Gavin A. Cloherty[a,b], Michael G. Berg[a,b]

[a] Infectious Disease Research, Abbott Diagnostics Division, Abbott Laboratories, Abbott Park, Illinois, USA.

[b] Abbott Pandemic Defense Coalition (APDC), Abbott Park, Illinois, USA.

[c] Department of Microbiology, Immunology and Transplantation, Laboratory of Clinical and Evolutionary Virology, Rega Institute, KU Leuven, Leuven, Belgium.

Address correspondence to Lester J. Perez

lester.perez@abbott.com

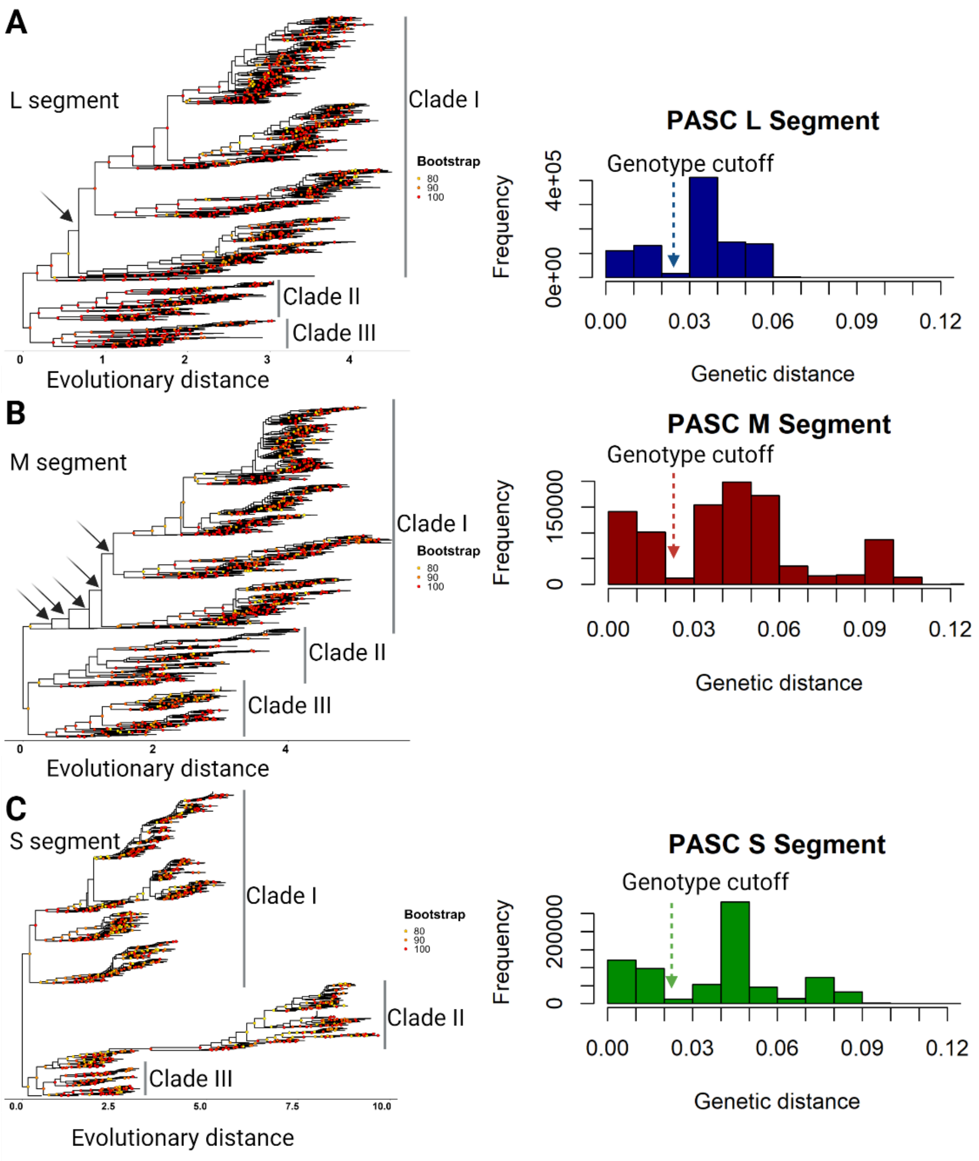

Figure S1. Topological and genetic reconciliation for all three segments of Severe Fever with Thrombocytopenia Syndrome Virus. A-C (left panels) Maximum likelihood tree for all non-redundant genomes available in GenBank for L, M and S segments, reconstructed to evaluate the lineage diversification and internal node support. Bootstrap values are scaled in color from 70% (yellow) to 100% (red); values <70% are not displayed. The three main clades supported for each segment were denoted. The arrows in the phylogenetic tree for L and M segment highlight the lack of the support of the nodes. **A-C, (right panels)** Representation of PAirwise Sequence Comparison (PASC) results obtained from the frequency distribution of pairwise distances for all (Supplementary Material Table S1-3) sequences using the SDT analysis. Cut-off values for each lower taxonomical level are indicated, denoting groupings of the same genotype level at 2% within the SFTSV species.

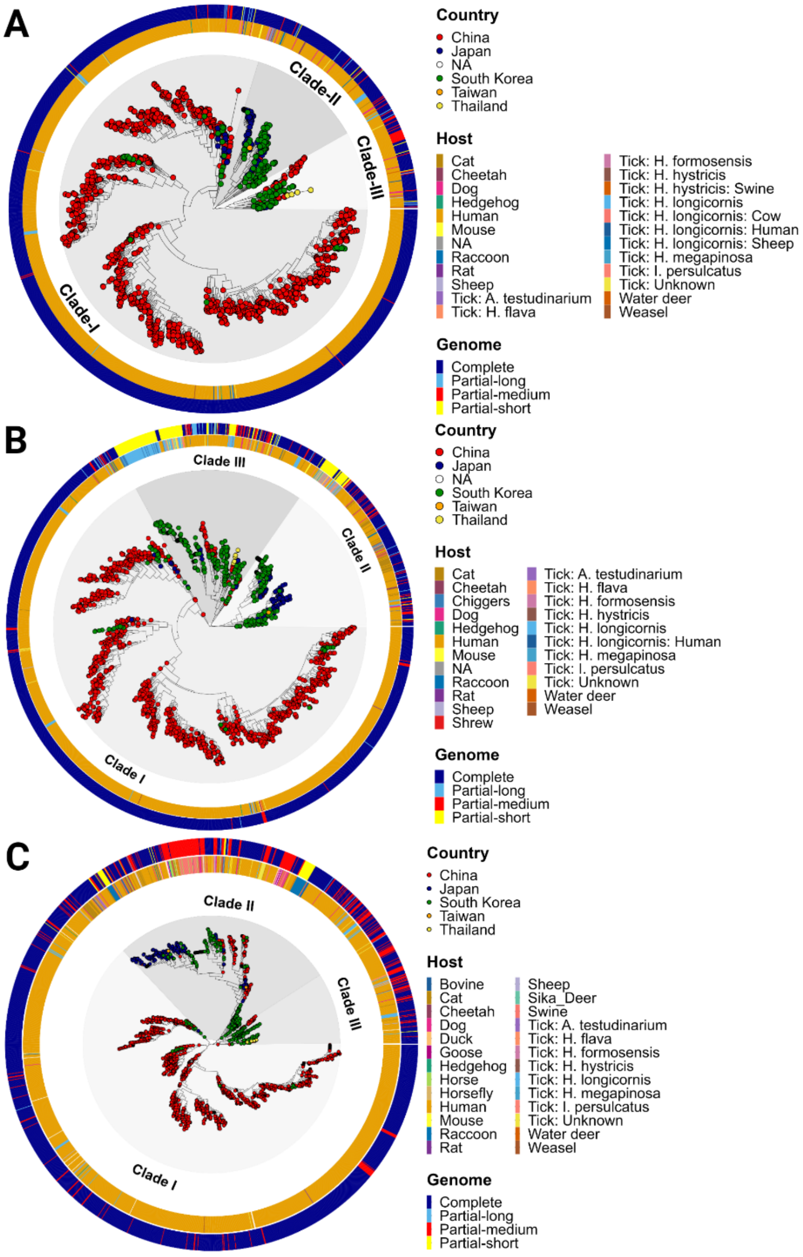

Figure S2. Phylogenetic analysis of SFTSV genomic segments. Maximum likelihood trees represent the phylogeny of all non-redundant SFTSV genomes in GenBank for segments A) L, B) M, and C) S. After the **PASC** and lineage demarcation (see **Figure S1**), we identified three primary clades per segment. Location of the strains isolation (countries) are color-coded at the tips, while the inner and outer circles indicate the isolation host and the influence of sequence length on phylogeny, respectively. In all cases the data was integrated using the *ggtreeExtra* R package.

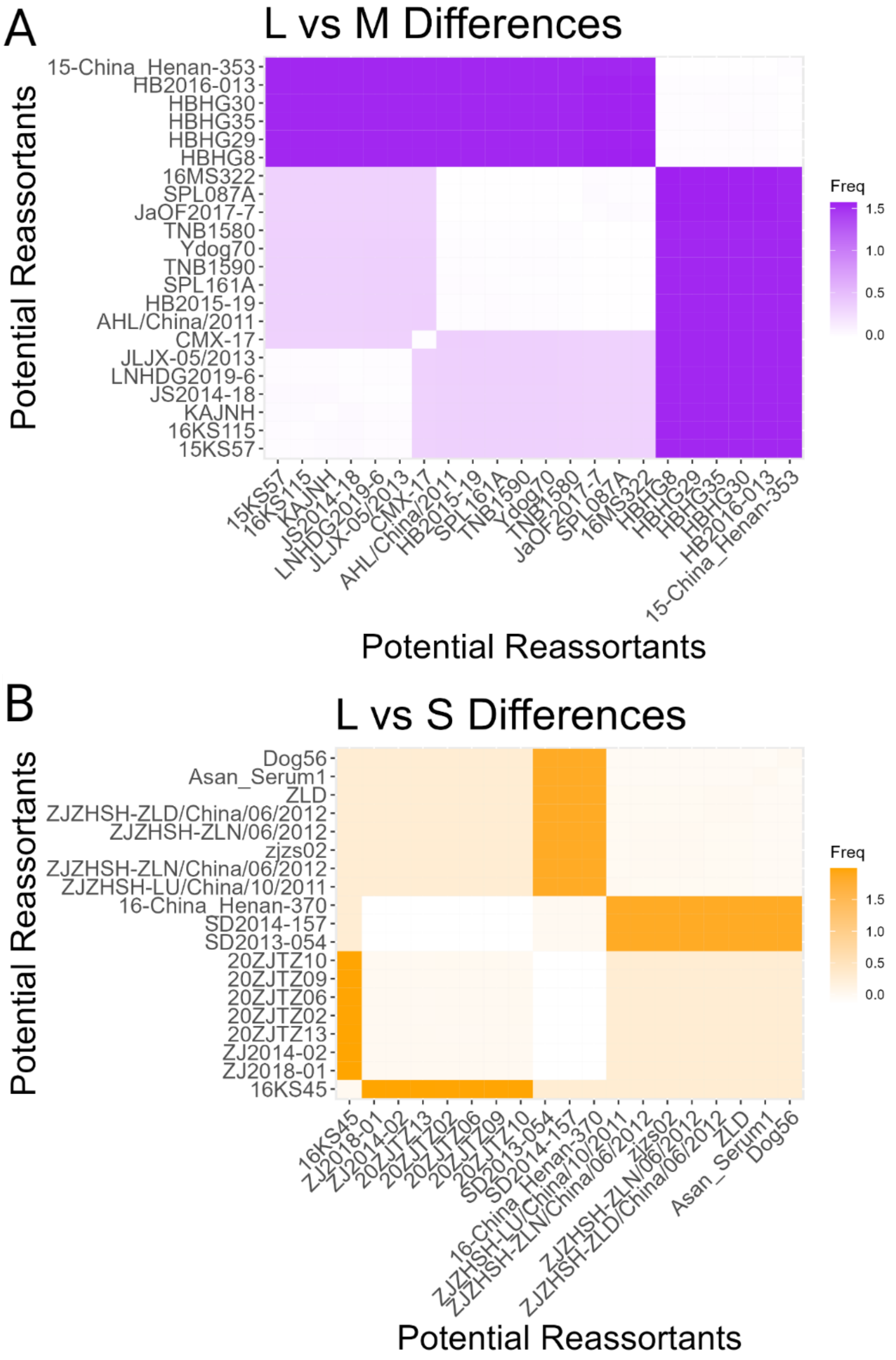

Figure S3. Identification of topological discrepancies in SFTSV strains by tanglegram analysis. The figure presents heatmaps that quantify genetic distances, calculated based on the frequency of desegregation among strains in the maximum likelihood (ML) trees. These heatmaps facilitate the comparison of topological congruence across different genomic segments, A) L segment versus M segment, B) L segment versus S segment.

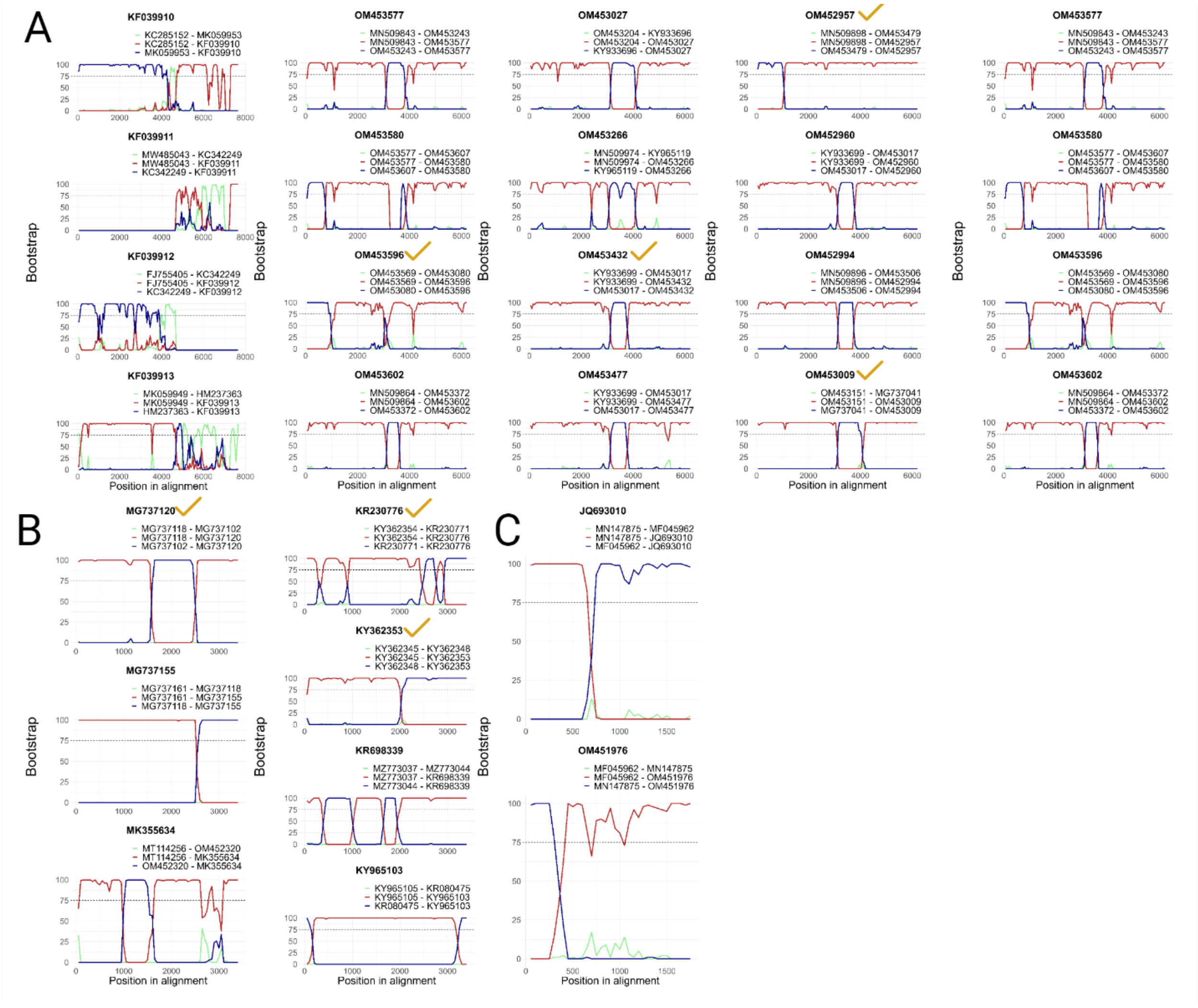

Figure S4. Recombination events in SFTSV Genomic Segments. Recombination events within the A) L, B) M, and C) S segments of the SFTSV genome were identified by the RDP5v5 software. Each event has been verified by at least three independent detection methods, ensuring a high level of confidence with statistical significance set at p< 0.01 post-Bonferroni correction. For ease of interpretation, the figure presents only those Bootscan results that demonstrate distinct recombination breakpoints with bootstrap support ≥75% threshold. Those recombinants confirmed by the temporal analysis of their parental strains (**FigureS5 and S6**) were denoted with yellow checkmarks.

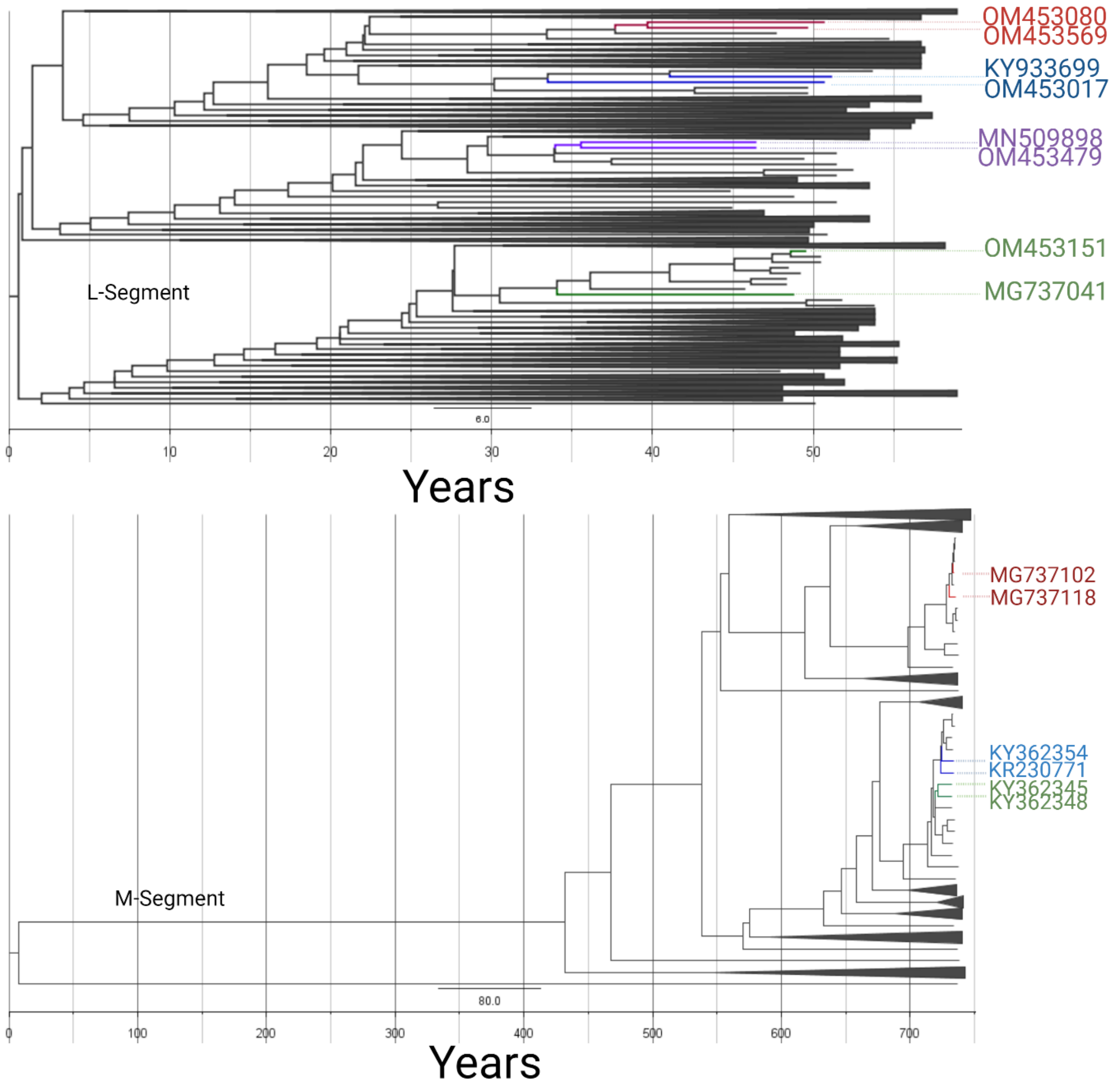

Figure S5. Temporal evaluation of parental strains for identified recombinants. The panels show MCC trees for the L segment (top) and M segment (bottom), identifying the ancestral origins and circulation epochs of the parental strains for each identified recombinant (**Supplementary Information FigureS4).** Only parental strains that emerged from a close ancestor (less than 10 branches) and circulated within 2.4 years (based on HPD95% of the most external node) of each other are included. Parental pairs are indicated with matching colors on the branches. Remaining clades are collapsed to improve clarity.

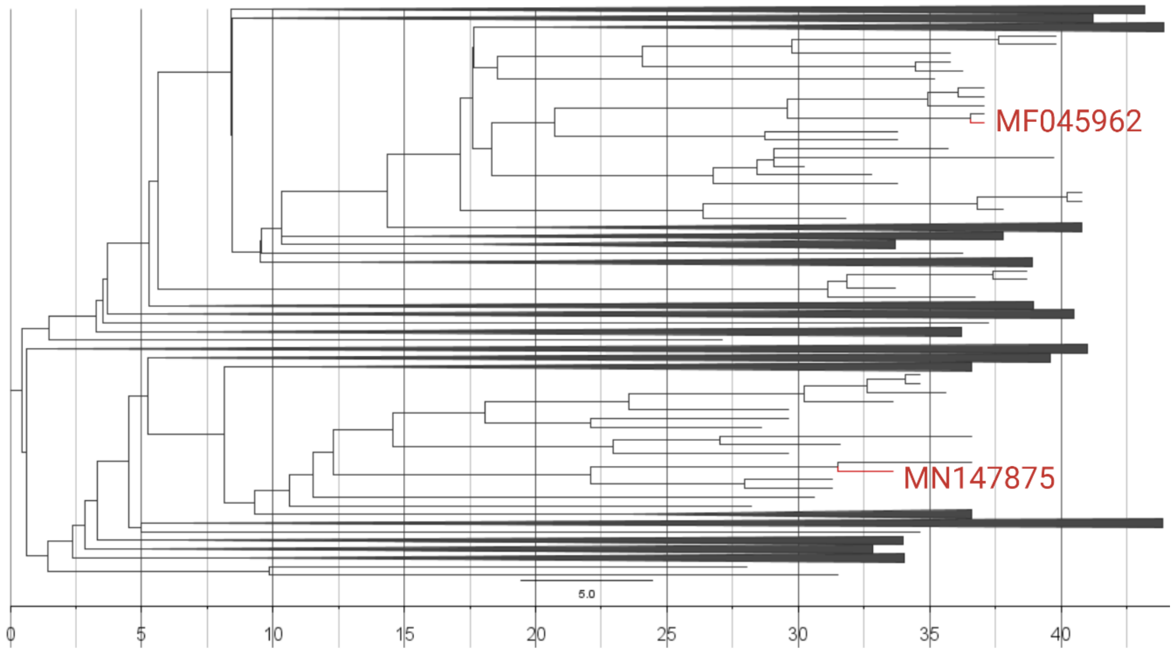

Figure S6. Temporal evaluation of parental strains for identified recombinants in Segments. MCC trees for the S is shown displaying also the ancestral origins and circulation epochs of the parental strains of both identified recombinants (**Supplementary Information FigureS4).** The branches of the parental strains are denoted in red, since they emerged in very distant ancestor and circulated in different temporal epoch both reassortant strains detected for the S segment were considered false positives.

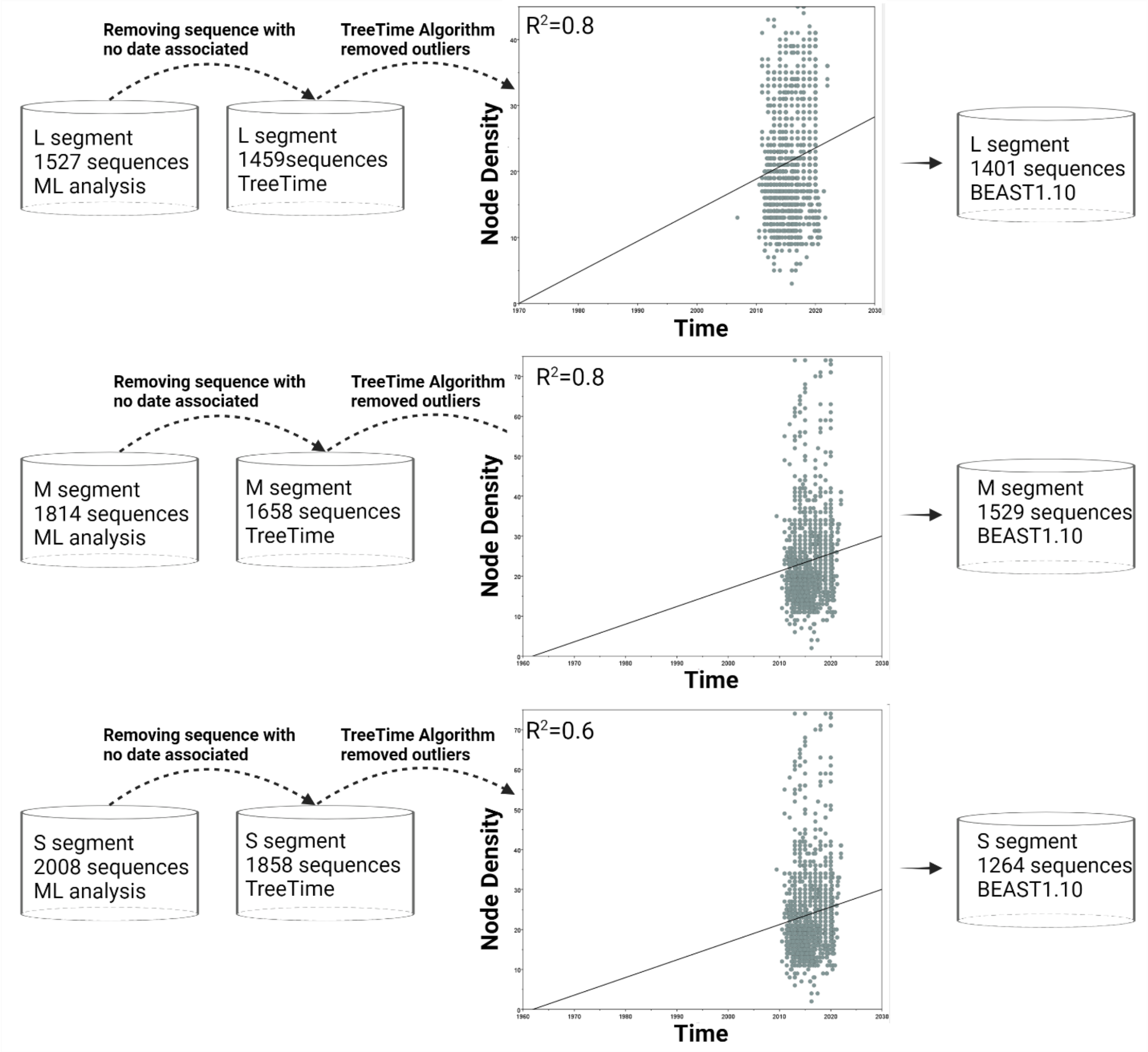

Figure S7. Workflow of data curation and molecular clock evaluation for all the tree genomic segments of SFTSV. The initial number of sequences and methodology used to evaluate outliers, the molecular clock, R^2 values and final number of sequences to be used in the BEAST analyses are shown.

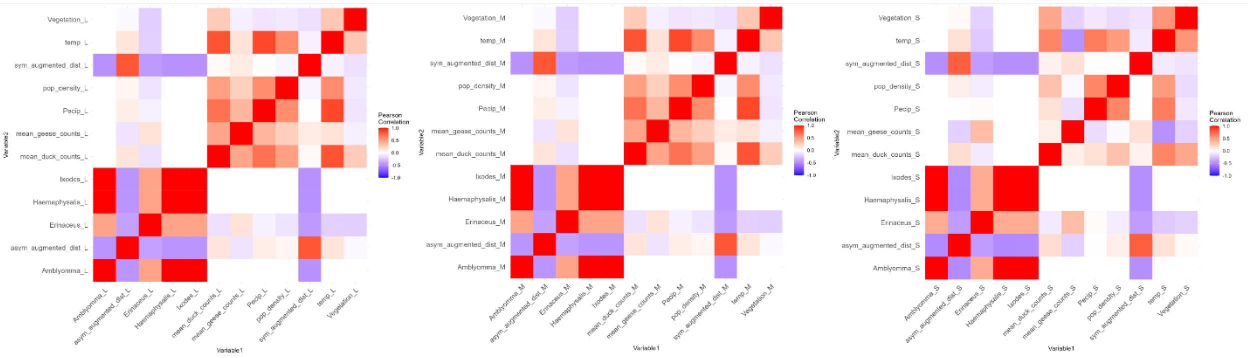

Figure S8. Analysis of collinearity for the predictors used in the GLM phylogeographic analyses. The different predictors included in the study (Extended Data Table 2) were evaluated for colinear effects, represented here by a color-coded matrix from left to right (L segment, M segment and S segment). Red and blue indicate positive and negative correlations, respectively.

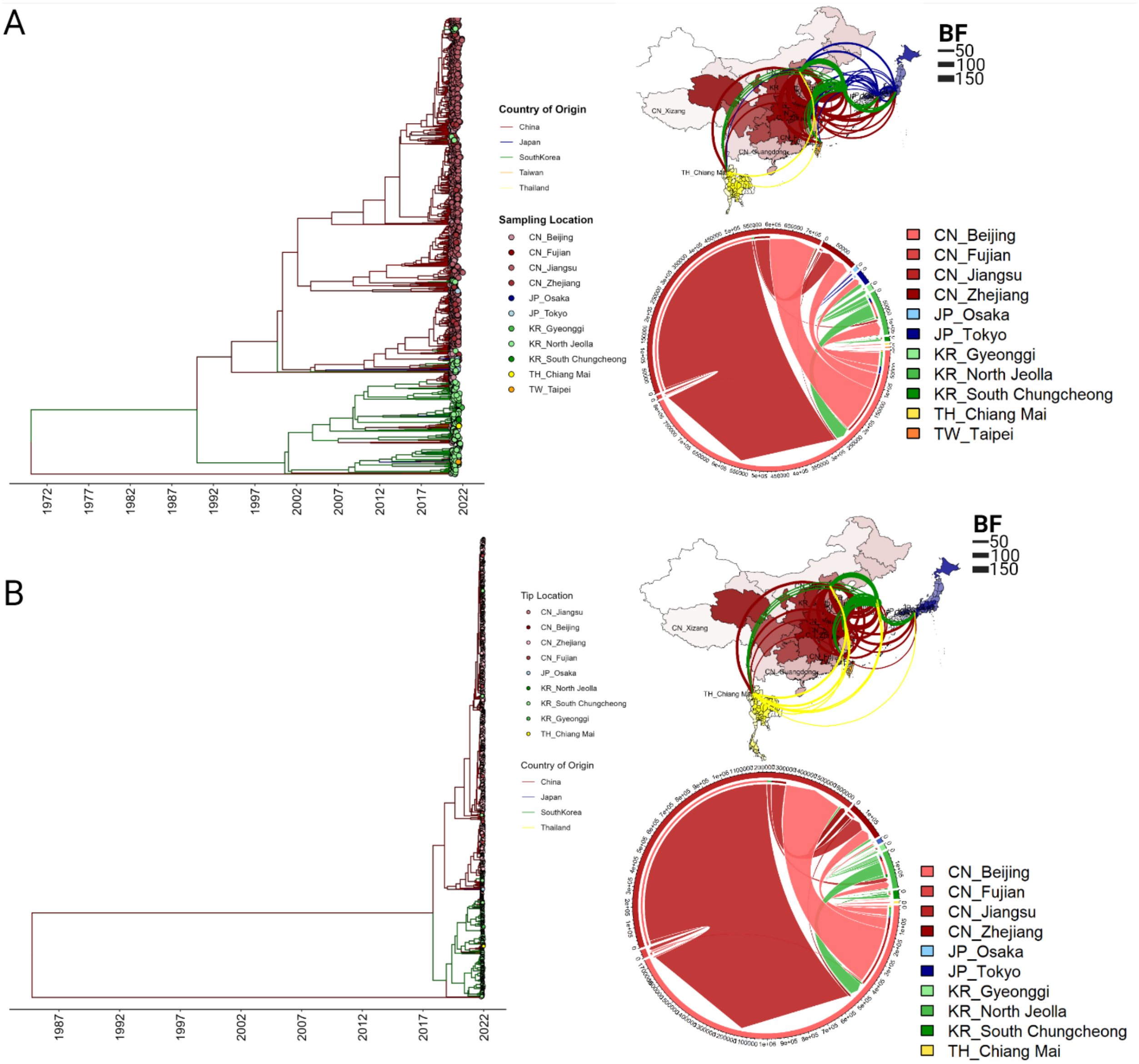

Figure S9. GLM Phylogeographic Analysis of SFTSV Spread for A (M segment) B (S segment). For both segments, Maximum Clade Credibility (MCC) trees identify South Korea as the likely origin of SFTSV proliferation. The analysis also identifies two predominant lineages: L1, with a significant presence in China, indicated by red, and L2, with significant dissemination in South Korea, denoted by green. The dynamic pathways of SFTSV geographical movement are denoted by Markov jump mappings. Only those transitions supported by a Bayes Factor > 20 are indicated, with Markov jump events represented in a circular layout using the *circlize* package in R.

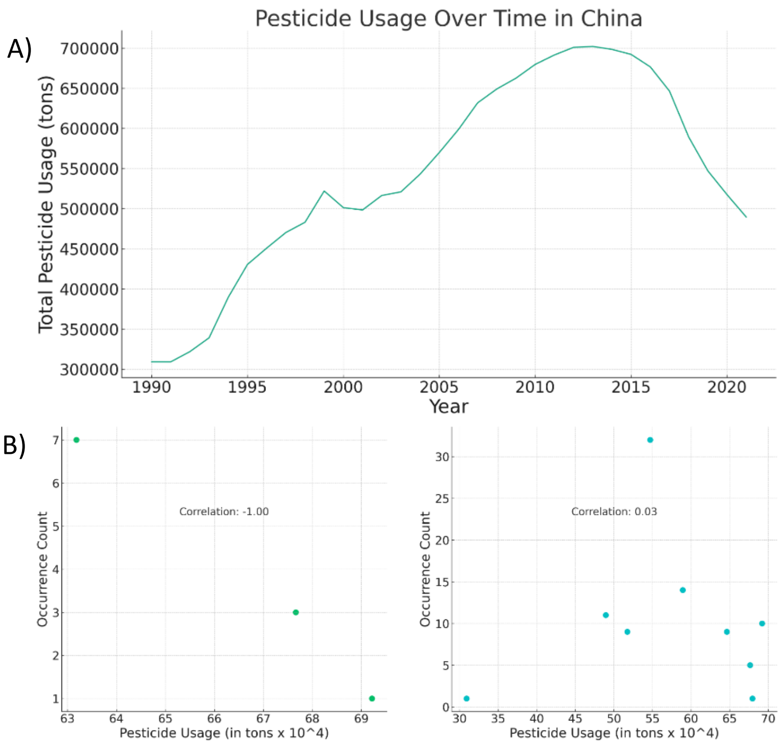

Figure S10. Impact of pesticide usage in tick populations in China. A) panel illustrates the temporal usage of pesticides in China from 1990 to 2020. B) panel shows the correlation of pesticide usage versus *H. hystricis* (left) and *H. longicornis* (right) occurrence.

Table S1. Assessment of Temporal Signals in all the tree genomic using BETS analysis.

Log marginal likelihood values for each genomic segment, obtained through path sampling and stepping-stone sampling methods. The presence of a temporal signal was quantified by the log Bayes factor, calculated from the difference in log marginal likelihoods between the heterochronous model (incorporating sampling dates) and the isochronous model (excluding sampling dates).

	Clock prior: URL Exponential distribution, mean=1.0						Clock prior: strict clock, coalescence Exponential distribution, mean=1.0					
	Path Sampling			Stepping-Stone Sampling			Path Sampling			Stepping-Stone Sampling		
	L	M	S	L	M	S	L	M	S	L	M	S
log marginal likelihood, isochronous model	-122744.7319	-81844.8739	-37442.88256	-122795.6651	-81900.8267	-37477.2278	-123025.7979	-82067.2013	-37295.9206	-123088.704	-82129.6486	-37332.49233
log marginal likelihood, heterochronous model	-122492.3451	-81356.0152	-37409.53595	-122553.2497	-81462.6899	-37447.80447	-122812.610	-81773.921	-37280.97317	-122863.246	-81831.3658	-37309.53595
log Bayes factor	**252.387**	**488.859**	**33.347**	**242.415**	**438.137**	**29.423**	213.188	293.280	14.947	225.458	298.283	22.956
Temporal signal	Yes	Yes	Yes	Yes	Yes	Yes	Yes	Yes	Yes	Yes	Yes	Yes

Table S2. Predictors included in the phylogeographic analysis using the GLM model.

Predictor category	Abbreviation	Description
Geographic	sym augmented dist	Haversine distances between the locations' population centroids, log-transformed, standardized
Geographic	asym augmented dist	Haversine distances between the locations' population centroids, log-transformed, standardized
Demographic	Population density	Origin/Destination population density, log-transformed, standardized
Climatic	Precipitation	Origin/Destination average precipitation / year
Climatic	Vegetation	Origin/Destination average vegetation / year
Climatic	Temperature	Origin/Destination average temperature / year
Ecological	Ducks movement	Origin/Destination average movent of Duck / year
Ecological	Geese movement	Origin/Destination average movent of Geese / year
Ecological	Ixodes	Augmented matrix of occurrence of Ixodes presulcatus
Ecological	Amblyomma	Augmented matrix of occurrence of Amblyomma testudinarium
Ecological	Haemaphysalis	Augmented matrix of joined occurrence of Haemaphysalis hystricis, H. formosenssi, H. flava, and H. longicornis
Ecological	Erianceus	Augmented matrix of occurrence of Erinaceus amurensis

Table S3. *Post-hoc* evaluation revealed of both SFTSV lineages using *Seraphim* R package

**Lineage**	**weighted diffusion coefficient [95%HPD] km**	**weighted branch dispersal velocity [95%HPD] km2/year**
Lineage 1	2072.638 [1927.300, 2352.748]	67.72596 [38.02244, 314.5962]
Lineage 2	871.8791 [589.9262, 2166.722]	15.6224 [9.500189, 54.79309]

Table S4. Impact of different environmental variables on the dispersal location of both lineages L1 and L2 of SFTSV in the Asia Region. Bayes factors (BF) supporting the association between environmental variables and tree node locations are shown. Each environmental variable was tested as a factor of attraction ("A") or repulsion ("R") for the viral lineages. We only consider a BF value >20 as strong support for a significant correlation between the environmental distances and dispersal durations (in bold).

Lineage	Environmental factor	BF Statistic E	BF Statistic E
L1	Elevation(A)	0	13.2857
	Vapor water(meassure of humidity)(A)	**997**	**165.6667**
	Precipitation(A)	**57.7059**	**23.3902**
	Solar radiation(A)	37.3846	1.3364
	Temperature(A)	**900**	**199**
	Vegetation(A)	900	9.2041
	Wind speed(A)	0.0142	6.5188
	Elevation(R)	900	0.0787
	Vapor water(meassure of humidity)(R)	0	0.007
	Precipitation(R)	0.0331	0.0471
	Solar radiation(R)	0.0374	0.6529
	Temperature(R)	0	0.004
	Vegetation(R)	0	0.0977
	Wind speed(R)	89.7273	0.1364
L2	Elevation(A)	0.0111	12.5135
	Vapor water(meassure of humidity)(A)	0.1378	1.7701
	Precipitation(A)	0.3008	3.4444
	Solar radiation(A)	0.0395	2.2154
	Temperature(A)	1.5747	2.1153
	Vegetation(A)	0.0142	1.079
	**Wind speed(A)**	**26.0352**	**23.1667**
	Elevation(R)	82.25	0.0695
	Vapor water(meassure of humidity)(R)	7.6121	0.4599
	Precipitation(R)	3.3624	0.23
	Solar radiation(R)	36	0.3928
	Temperature(R)	0.5807	0.4104
	Vegetation(R)	75.8462	0.9194
	Wind speed(R)	0.1809	0.2626

Table S5. Impact of different environmental variables on the dispersal velocity of both lineages L1 and L2 of SFTSV in the Asia Region. Positive, univariate linear regression coefficients and supporting Q-values were estimated; only values of LR and Qs > 95 were considered for randomization analyses (in bold).

Lineage	Environmental factor	Sum(LR >0)	Sum (Qs>0)
L1	Elevation(C)	99.9	85.6
	**Vapor water(meassure of humidity)(C)**	**99.9**	**97.8**
	**Precipitation(C)**	**99.9**	**99**
	**Solar radiation(C)**	**99.9**	**98.1**
	**Temperature(C)**	**99.9**	**99**
	**Vegetation(C)**	**99.9**	**97.8**
	**Wind speed(C)**	**99.9**	**99.5**
	Elevation(R)	99.9	0.2
	Vapor water(meassure of humidity)(R)	99.9	0
	Precipitation(R)	99.9	0
	Solar radiation(R)	99.9	0.2
	Temperature(R)	99.9	0.1
	Vegetation(R)	99.9	0.1
	Wind speed(R)	100	2.6
L2	Elevation(C)	27.2	55.6
	Vapor water(meassure of humidity)(C)	24.1	38.5
	Precipitation(C)	27.3	61
	Solar radiation(C)	27.6	53.4
	Temperature(C)	27.9	55.4
	Vegetation(C)	26.5	82.1
	Wind speed(C)	27.2	55.6
	Elevation(R)	26	51.3
	Vapor water (measure of humidity)(R)	24.1	38.5
	Precipitation(R)	27.3	61
	Solar radiation(R)	27.6	53.4
	Temperature(R)	27.9	55.4
	Vegetation(R)	26.5	82.1
	Wind speed(R)	27.2	55.6

Table S6. Randomization evaluation on the impact of different environmental variables that yielded statistical support in Extended Data Table 4 on the dispersal velocity of lineage L1 of SFTSV in the Asia Region. Only BF > 20 were considered with statistical support (in bold).

**Enviromental factor**	**Sum(LR >0)**	**Sum (Qs>0)**	**BF randomization=1**
Wind speed(C)	99.9	99.5	6.874
**Vegetation(C)**	**99.9**	**97.8**	**51.6316**
**Temperature(C)**	**99.9**	**99**	**21.2222**
**Solar radiation(C)**	**99.9**	**98.1**	**37.4615**
**Precipitation(C)**	**99.9**	**99**	**34.7143**
Vapor water (measure of humidity)(C)	99.9	97.8	5.6667

Table S7. Rescaling analysis based on the environmental variables detected as potential impactors on the dispersal velocity of lineage L1 of SFTSV. Analysis was performed as described previously in Dellicour (2023) following the expression vR = 1 + k * v0, where k is the scaling factor and v0 is the raster for the variable assessed. Only a BF value >20 was considered strong support for significant correlation in at least two rescaled rasters of the environmental variable.

Enviromental factor	k	Sum(LR >0)	Sum (Qs>0)	BF value
	10	99.9	99.3	8.1743
**Vegetation(C)**	100	**99.9**	**98.6**	**28.4118**
	1000	**99.9**	**98.1**	**82.3333**
	10	99.9	99.9	9
Temperature(C)	100	99.9	99	12.6986
	1000	99.9	99	15.3934
	10	99.9	99	9
**Solar radiation(C)**	100	**99.9**	**98.7**	**61.5**
	1000	**99.9**	**98.3**	**40.6667**
	10	99.9	76.5	1.2676
Precipitation(C)	100	99.9	86.9	4.291
	1000	99.9	98.5	17.1818

Evaluation of predictors

Segment	Predictor	Inclusion Probability	# Predictors	Prior Odds	Posterior Odds	Bayes Factor
L	Avg_geese_count_origin	0.000565691	7	0.104089514	0.000566011	0.005
L	Avg_geese_count_destination	0.006222599	7	0.104089514	0.006261563	0.06
L	Amblyomma	0.986140574	7	0.104089514	71.15306122	683.576
L	Erinaceus	0.994343092	7	0.104089514	175.775	1688.691
L	Avg_Precipitation_destination	1	7	0.104089514	99999	960701.962
L	Avg_Precipitation_origin	1	7	0.104089514	99999	960701.962
L	Haemaphysalis	1	7	0.104089514	99999	960701.962
M	Avg_geese_count_destination	0	10	0.071773	0	0
M	Avg_geese_count_origin	0	10	0.071773	0	0
M	Population_origin	0	10	0.071773	0	0
M	Sym_augmented_dist	0.02732644	10	0.071773463	0.028094153	0.391
M	Erinaceus	0.038404727	10	0.071773463	0.039938556	0.556
M	Amblyomma	0.947562777	10	0.071773463	18.07042254	251.77
M	Avg_Precipitation_destination	1	10	0.071773463	99999	1393258.685
M	Avg_Precipitation_origin	1	10	0.071773463	99999	1393258.685
M	Haemaphysalis	1	10	0.071773463	99999	1393258.685
M	Population_destination	1	10	0.071773463	99999	1393258.685
S	Avg_duck_count_destination	0	14	0.050757	0	0
S	Avg_geese_count_origin	0	14	0.050757	0	0
S	Population_Density_destination	0	14	0.050757	0	0
S	Population_Density_origin	0	14	0.050757	0	0
S	Vegetation_Index_destination	0	14	0.050757	0	0
S	Vegetation_Index_origin	0	14	0.050757	0	0
S	Avg_geese_count_destination	0.00226886	14	0.050756639	0.002274019	0.045
S	Avg_Temperature_origin	0.002836075	14	0.050756639	0.002844141	0.056
S	Avg_Temperature_destination	0.00340329	14	0.050756639	0.003414912	0.067
S	Avg_duck_count_origin	0.097135564	14	0.050756639	0.10758599	2.12
S	Erinaceus	0.513471356	14	0.050756639	1.055377441	20.793
S	Avg_Precipitation_destination	1	14	0.050756639	99999	1970165.926
S	Avg_Precipitation_origin	1	14	0.050756639	99999	1970165.926
S	Haemaphysalis	1	14	0.050756639	99999	1970165.926

Inclusion probabilities, prior odds, posterior odds and Bayes Factors for all predictors used in analyses using the non collinear set of predictors.

Segment	Predictor	Inclusion Probability	# Predictors	Prior Odds	Posterior Odds	Bayes Factor
L	Avg_duck_count_destination	0.0	4	0.189207	0.0	0.0
L	Avg_Temperature_destination	1.0	4	0.18920711500272114	99999.0000004551	528516.066
L	Avg_Temperature_origin	1.0	4	0.18920711500272114	99999.0000004551	528516.066
L	Avg_duck_count_origin	1.0	4	0.18920711500272114	99999.0000004551	528516.066
M	Avg_duck_count_origin	0.0	6	0.122462	0.0	0.0
M	Ixodes	0.013747054202670856	6	0.12246204830937302	0.013938669852648347	0.114
M	Avg_Temperature_destination	0.7085624509033779	6	0.12246204830937302	2.4312668463611864	19.853
M	Avg_duck_count_destination	0.9756480754124115	6	0.12246204830937302	40.06451612903204	327.159
M	Asym_augmented_dist	1.0	6	0.12246204830937302	99999.0000004551	816571.349
M	Avg_Temperature_origin	1.0	6	0.12246204830937302	99999.0000004551	816571.349
S	Asym_augmented_dist	0.9059973452395318	4	0.18920711500272114	9.637997432605905	50.939
S	Amblyomma	1.0	4	0.18920711500272114	99999.0000004551	528516.066
S	Ixodes	1.0	4	0.18920711500272114	99999.0000004551	528516.066
S	Sym_augmented_dist	1.0	4	0.18920711500272114	99999.0000004551	528516.066

Inclusion probabilities, prior odds, posterior odds and Bayes Factors for all predictors used in analyses using the collinear set of predictors.

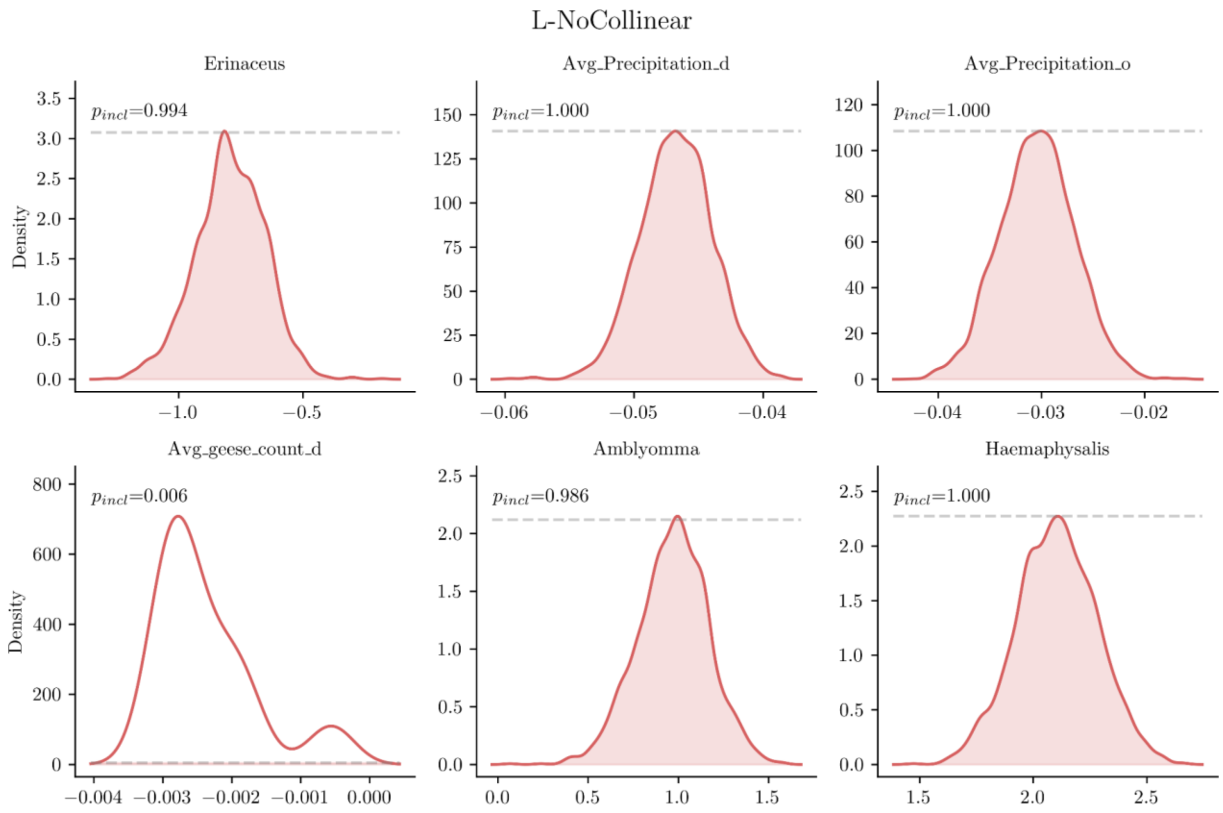

Kernel density plots of the posterior conditional effect size of each predictor used in the segment L non collinear analysis. The inclusion probabilities of each predictor are represented by the vertical shading of each density plot, with an inclusion probability of 1 corresponding to the complete area under a curve being shaded. The density plot for the Average Geese Count at the Origin predictor is not included as it was never included in the model.

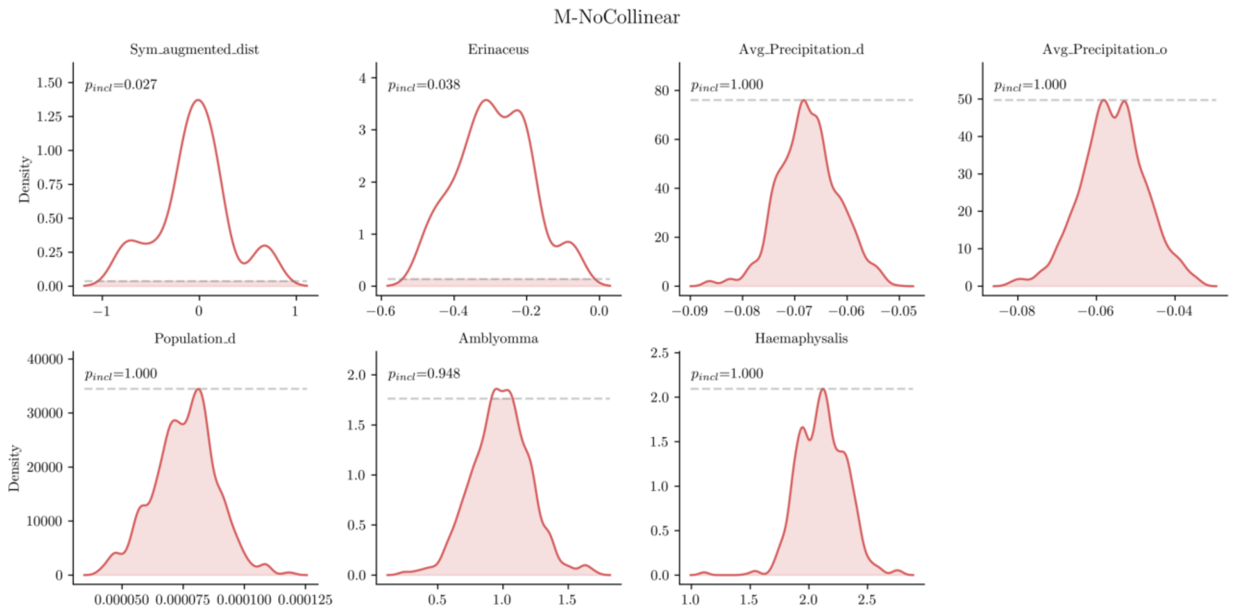

Kernel density plots of the posterior conditional effect size of each predictor used in the segment M non collinear analysis. The inclusion probabilities of each predictor are represented by the vertical shading of each density plot, with an inclusion probability of 1 corresponding to the complete area under a curve being shaded. The following predictors were never included in the model and thus have no density curves: Average Geese Count at Origin, Average Geese Count at Destination, and Population Density at Origin.

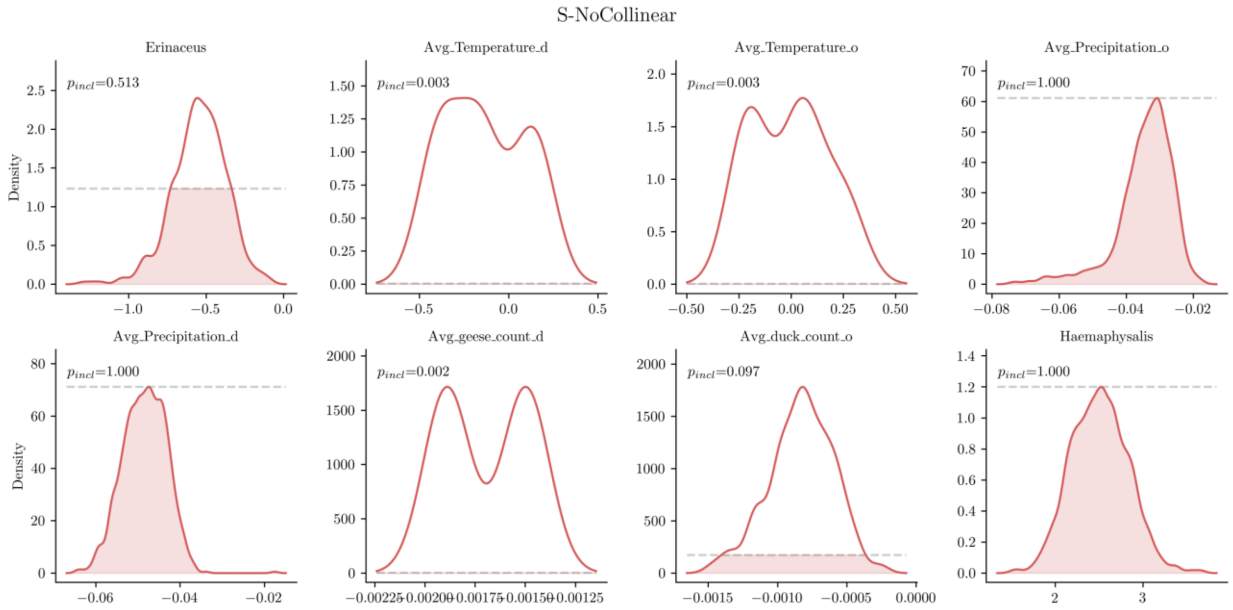

Kernel density plots of the posterior conditional effect size of each predictor used in the segment S non collinear analysis. The inclusion probabilities of each predictor are represented by the vertical shading of each density plot, with an inclusion probability of 1 corresponding to the complete area under a curve being shaded. The following predictors were never included in the model and thus have no density curves: Average Geese Count at Origin, Average Duck Count at Destination, and Population Density at Origin, Population Density at Destination, Vegetation Index at Origin, and Vegetation Index at Destination.

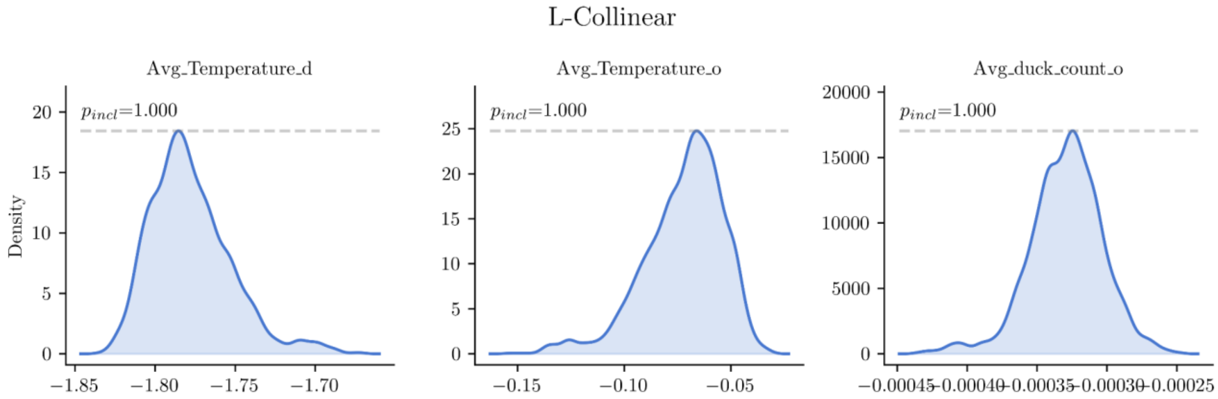

Kernel density plots of the posterior conditional effect size of each predictor used in the segment L collinear analysis. The inclusion probabilities of each predictor are represented by the vertical shading of each density plot, with an inclusion probability of 1 corresponding to the complete area under a curve being shaded. The density plot for the Average Duck Count at the Destination predictor is not included as it was never included in the model.

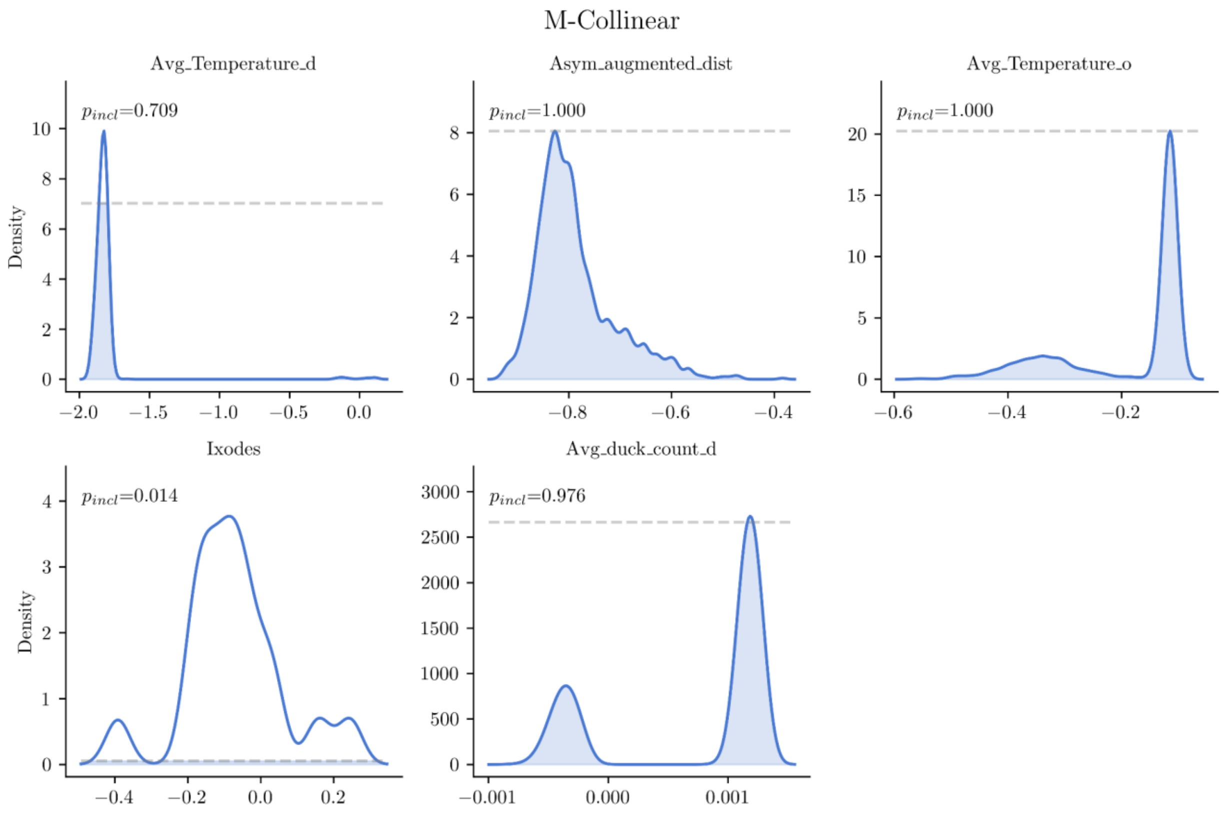

Kernel density plots of the posterior conditional effect size of each predictor used in the segment L collinear analysis. The inclusion probabilities of each predictor are represented by the vertical shading of each density plot, with an inclusion probability of 1 corresponding to the complete area under a curve being shaded. The density plot for the Average Duck Count at the Origin predictor is not included as it was never included in the model.

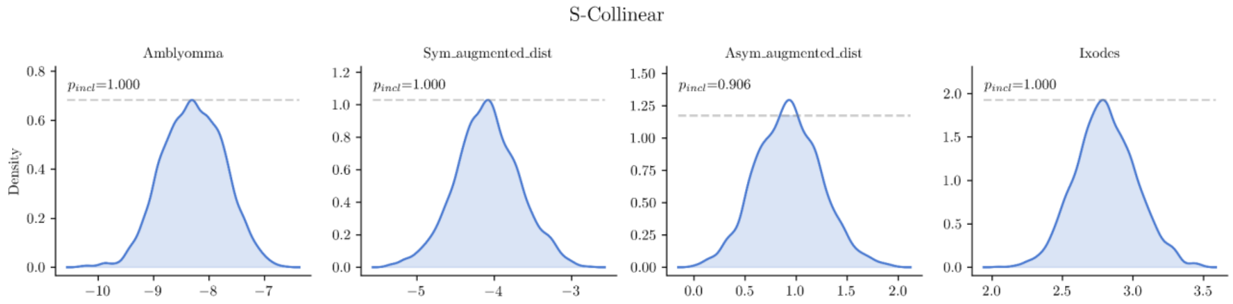

Kernel density plots of the posterior conditional effect size of each predictor used in the segment L collinear analysis. The inclusion probabilities of each predictor are represented by the vertical shading of each density plot, with an inclusion probability of 1 corresponding to the complete area under a curve being shaded.
